# Linker histones are fine-scale chromatin architects modulating developmental decisions in *Arabidopsis*

**DOI:** 10.1186/s13059-019-1767-3

**Published:** 2019-08-07

**Authors:** Kinga Rutowicz, Maciej Lirski, Benoît Mermaz, Gianluca Teano, Jasmin Schubert, Imen Mestiri, Magdalena A. Kroteń, Tohnyui Ndinyanka Fabrice, Simon Fritz, Stefan Grob, Christoph Ringli, Lusik Cherkezyan, Fredy Barneche, Andrzej Jerzmanowski, Célia Baroux

**Affiliations:** 1 Institute of Plant and Microbial Biology, Zürich-Basel Plant Science Center, University of Zürich, Zürich, Switzerland; 2 Institute of Biochemistry and Biophysics, Polish Academy of Sciences, Pawinskiego 5a, 02-106 Warsaw, Poland; 3 Department of Molecular, Cellular & Developmental Biology, Yale University, 352a Osborn memorial laboratories, New Haven, CT, 06511 USA; 4 Département de Biologie, IBENS, Ecole Normale Supérieure, CNRS, INSERM, PSL Research University, 46 rue d’Ulm, F-75005 Paris, France; 5 College of Inter-Faculty Individual Studies in Mathematics and Natural Sciences, University of Warsaw, 02-089 Warsaw, Poland; 6 Department of Biomedical Engineering, Northwestern University, Evanston, IL 60208 USA; 7 Faculty of Biology, University of Warsaw, Pawinskiego 5a, 02-106 Warsaw, Poland

**Keywords:** Linker histones, H1, Chromatin, Heterochromatin, Histone methylation, Developmental transitions, Nucleosome occupancy

## Abstract

**Background:**

Chromatin provides a tunable platform for gene expression control. Besides the well-studied core nucleosome, H1 linker histones are abundant chromatin components with intrinsic potential to influence chromatin function. Well studied in animals, little is known about the evolution of H1 function in other eukaryotic lineages for instance plants. Notably, in the model plant *Arabidopsis*, while H1 is known to influence heterochromatin and DNA methylation, its contribution to transcription, molecular, and cytological chromatin organization remains elusive.

**Results:**

We provide a multi-scale functional study of *Arabidopsis* linker histones. We show that H1-deficient plants are viable yet show phenotypes in seed dormancy, flowering time, lateral root, and stomata formation—complemented by either or both of the major variants. H1 depletion also impairs pluripotent callus formation. Fine-scale chromatin analyses combined with transcriptome and nucleosome profiling reveal distinct roles of H1 on hetero- and euchromatin: H1 is necessary to form heterochromatic domains yet dispensable for silencing of most transposable elements; H1 depletion affects nucleosome density distribution and mobility in euchromatin, spatial arrangement of nanodomains, histone acetylation, and methylation. These drastic changes affect moderately the transcription but reveal a subset of H1-sensitive genes.

**Conclusions:**

H1 variants have a profound impact on the molecular and spatial (nuclear) chromatin organization in *Arabidopsis* with distinct roles in euchromatin and heterochromatin and a dual causality on gene expression. Phenotypical analyses further suggest the novel possibility that H1-mediated chromatin organization may contribute to the epigenetic control of developmental and cellular transitions.

**Electronic supplementary material:**

The online version of this article (10.1186/s13059-019-1767-3) contains supplementary material, which is available to authorized users.

## Background

Linker histones (H1) belong to the major constituents of plant and animal chromatin besides the core nucleosomal histones. H1 variants (collectively referred to as H1 thereafter) appeared early during evolution as lysine-rich, proto-linker histones found in the ancestral eukaryotes such as protists but not Archaea [1]. In contrast to core nucleosomal constituents, H1 form a highly divergent class of histones [1]. H1 typically possesses a tripartite structure conserved across all eukaryotes: it consists of a short and flexible N-terminal tail, a structured globular domain (GH1) which interacts with a nucleosome dyad, and a structurally disordered, lysine-rich (highly basic) C-terminal tail. The C-terminal tail of H1 variants varies in length and composition among isotypes and organisms, conferring their various chromatin compaction potential by interacting with internucleosomal DNA and drawing adjacent nucleosomes together [2, 3]. In animals, several H1 isotypes can coexist in the same cell type playing both redundant and specific roles in chromatin organization [4]. H1 proteins constitute a highly mobile fraction of the animal chromatin; their apparent constitutive presence results from a steady-state level of dynamic binding [5]. H1-mediated higher-order chromatin organization influences RNA polymerase II accessibility, hence gene expression, DNA replication, chromosome segregation, and DNA repair [4, 6–9]. In *Drosophila*, *Neurospora crassa*, and mammalian cells, H1 deposition is part of a crosstalk with the epigenetic landscape, notably DNA methylation [10–12] and histone H3 methylation [12–14]. Given the profound impact of H1 proteins on chromatin organization, it is surprising that H1 depletion is tolerated in some organisms: this is the case of *Tetrahymena*, yeast, fungi, worms [15–19]. However, in mouse and *Drosophila*, it impairs viability [10, 20]. The intrinsic role of H1 in chromatin organization and yet the variable impact of its depletion create an apparent paradox which is difficult to address given the high functional redundancy between variants, particularly in mammalian genomes.

In the plant kingdom, H1 gene variants can be traced back to the earliest land plants [21]. The flowering plant *Arabidopsis thaliana* possesses only three canonical H1 variants, and the reduced number of isotypes makes it suitable for analyzing H1 functions [21, 22]. Two of them, H1.1 and H1.2, are ubiquitous in differentiated shoot and root tissues and are expressed throughout vegetative development. Whether they play distinct or similar roles on chromatin organization is not known. Despite their apparent ubiquitous expression, both variants are evicted at the somatic-to-reproductive cell fate transition and undetectable in the reproductive lineage [23–25]. Unlike in animals where germline-specific H1 variants have been described [26], no gametophyte-specific variants have yet been characterized in plants. H1.1 protein abundance is nevertheless restored in male gametes (Baroux, unpublished and [27]) where it could thus play a sperm cell-specific function, yet to be determined. H1.3, by contrast, is not a routine variant but is specifically incorporated in response to abiotic stresses: this stress-inducible variant is thought to act as a pioneer factor priming transcriptional reprogramming that leads to physiological adaptations [28]. The genomic distribution of H1 variants is widely present along the *Arabidopsis* genome, spanning both heterochromatin and euchromatin chromosomal domains [28, 29]. Specifically, H1.1 and H1.2 are enriched at the 3′ and 5′ ends of transposable elements (TEs) and over gene bodies where its profile is anti-correlated to the transcription and H3K4me3 levels [28]. They are subjected to numerous post-translational modifications with undescribed functions [30]. H1 variants further influence DNA methylation patterns in *Arabidopsis*, primarily but not exclusively, in heterochromatin and in all sequence contexts (i.e., CG, CHG, CHH) [22, 28, 31, 32]. H1-containing nucleosomes are thought to create a barrier to DNA methyltransferases, a structural conflict that can be resolved by the activity of SWI/SNF types of chromatin remodelers [32, 33].

Overall, however, the functional influence of H1 on chromatin organization and function in plants remains elusive, and our understanding of H1 function on cell specification and development is limited. Whereas H1 downregulation by RNAi knockdown in *Arabidopsis* produced pleiotropic phenotypes [22], where possible off-target effects on H1-related proteins cannot be excluded [21], double and triple knockout lines are viable and provide a unique opportunity to investigate the role of H1 during development [28, 31].

Here, we report that H1 has a profound role not only in heterochromatin but also in euchromatin in *Arabidopsis*, which was so far overlooked. It regulates chromatin compaction and organization at the nuclear level, being required for heterochromatin formation and influencing on the spatial distribution of nanoscale, compact domains in euchromatin. Mutant analyses reveal that H1 restricts nucleosome mobility and influences H3K9 acetylation, H3K27, and H3K4 methylation levels. We also show that H1 regulates nucleosome distribution and provides distinct nucleosome density over expressed genes correlating with their expression levels. Interestingly, these nucleosomal states and large-scale chromatin structures mediated by H1 are not epistatic to transcriptional regulation for a vast majority of loci. A small fraction of genes and TEs is, however, H1-sensitive suggesting that chromatin level regulation primes over other transcriptional controls for these loci. Finally, the analysis of mutant and complemented mutant lines unveils a new role of H1-mediated organization during development.

## Results

### H1-deficient *Arabidopsis* lines show deregulation of several developmental transitions

**Fig. 1:**
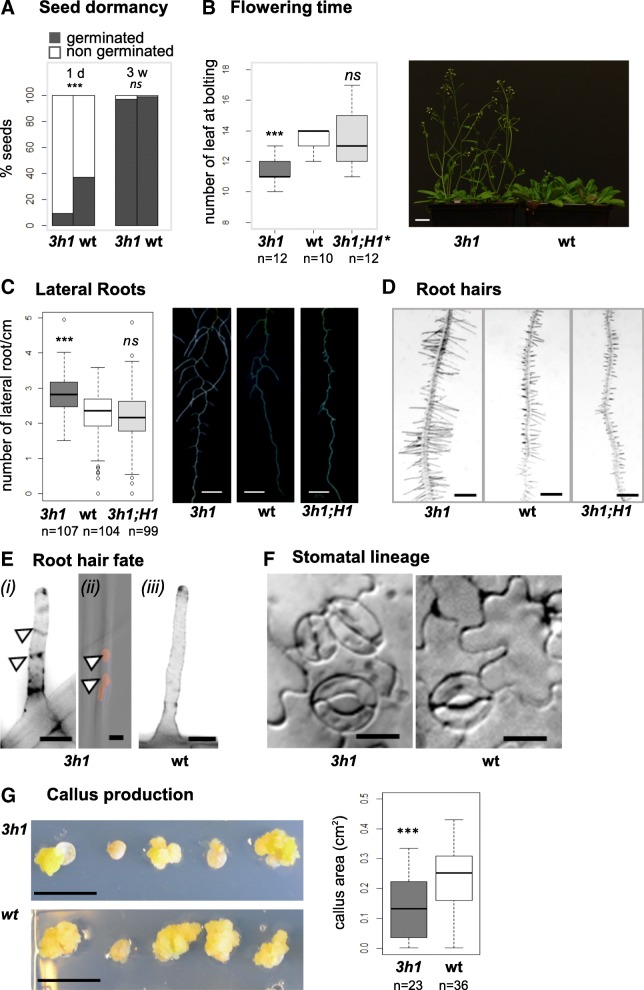
H1 depletion relaxes the epigenetic control of several developmental and cellular transitions. *3h1* mutant plants show a relaxed control of seed dormancy (**a**), flowering time (**b**), lateral root formation (**c**), root hair density (**d**), root hair fate (**e**), stomatal spacing (**f**), and are impaired in callus production in vitro. *3h1* shows, compared to wild-type **a** prolonged dormancy, i.e., lower germination rate 1 day post-harvest but alleviated 3 weeks post-harvest, **b** early flowering measured by the number of rosette leaves at bolting (*n* = number of plants analyzed in this replicate; more experiment replicates are shown in Additional file 1: Figure S1), **c** increased number of lateral roots (eight DAG seedlings), **d** increased root hair density (scale bar, 200 μm), **e** occasional multi-cellular root hairs showing visible cell walls (arrows) in *Renaissance* staining (i, iii) compared to in wild-type and additional nuclei in DAPI counterstaining (ii: arrows, red; additional examples and comments are presented in Additional file 1: Figure S2; scale bar, 10 μm), **f** stomatal complexes with reduced spacing (adaxial cotyledon epidermis; see Additional file 1: Figure S2 for additional examples and quantifications; scale bar, 20 μm), and **g** decreased callus size produced from excised cotyledons in vitro (scale bar, 1 cm). Wild-type segregants (wt) were compared with triple mutant tissues/seedlings (*3h1*) and, whenever indicated, with complemented lines expressing H1.1 and H1.2 variants only (*3h1*; H1) or all three H1 variants (*3h1*; H1*)*.* Statistical tests (**a**, **b** Welch *t* test; **c** Fisher exact test) were performed against wt replicates, ***p < 0.001; ns, not significant

T-DNA insertion mutant alleles for the three *H1* genes were previously introgressed producing double and triple mutants which are viable [25, 28, 31, 32]. This is in contrast to RNAi lines inducing pleiotropic aberrations [22], where possible off-target effects on H1-related genes [21] cannot be excluded. *H1* triple (T-DNA) mutant plants, thereafter called *3h1*, were previously characterized at the molecular level and do not show detectable levels of *H1* transcripts nor H1 proteins [25]. *3h1* appeared at first normal with regular shoot and root apparatus indicating that organogenesis coordinated by the apical meristems are not affected. We investigated the development of *3h1*-mutant plants in further details and noticed subtle phenotypes at developmental transitions. We noticed for example that a large fraction of *3h1* seeds underwent a prolonged dormancy after harvest (Fig. 1a). In addition, *3h1* plants showed precocious induction of flowering (Fig. 1b, Additional file 1: Figure S1), a phenotype which was rescued by the introduction of tagged H1 variants in *3h1* plants (Additional file 1: Figure S1C). We then rationalized that H1 might be implicated in other developmental transitions. To test this hypothesis, we analyzed the formation of lateral roots, root hairs, and epidermal stomata as traits reflecting the cell identity establishment concurrent to organogenesis. In *Arabidopsis*, the specification of lateral root primordia from pericycle founder cells and the differentiation of root hairs from epidermal cells follow a regular pattern modulated by developmental and environmental cues and subjected to epigenetic regulation [34, 35]. Firstly, compared to wild type, *3h1* seedlings produced a higher number of lateral roots per root length unit (Fig. 1c) and a higher density of root hairs (Fig. 1d). Again, both defects were rescued upon complementation with H1.1 and H1.2 transgenes (Fig. 1c, d; Additional file 1: Figure S2A). These observations suggest that H1 is required for proper spatial and temporal control of lateral root and root hair lineage initiation. In addition, the unicellular fate of root hairs was occasionally compromised in *3h1* but not in wild-type plants, with the appearance of multiple nuclei and cell boundaries (Fig. 1e, Additional file 1: Figure S2B). Interestingly, this trait was previously correlated with an unstable epigenetic root hair cell fate in mutants impaired in *Polycomb*-group Repressive Complex2 (PRC2)-based chromatin regulation [36].

Secondly, we noticed that stomata patterning in the epidermis of cotyledons was altered in *3h1* with a higher occurrence of high-degree (tertiary and quaternary) clusters, associated with complex arrangements, collated stomata, or atypical division patterns in early stages. These features were not found in the wild type and were restored upon the introduction of H1.1 and H1.2 transgenes (Fig. 1f, Additional file 1: Figure S2C). These observations suggest a loose control by H1 of stomatal spacing presumably involving occasional re-initiation events [37]. Finally, we also tested how *3h1* tissues respond to reprogramming in in vitro culture. We measured a decreased efficiency in callus development compared to the wild type (Fig. 1g), a feature mostly attributed to H1.3 in our complementation experiments (Additional file 1: Figure S3). It was shown before that for efficient leaf-to-callus transition, genome-wide reprogramming of H3K27me3 is a critical step, and in PRC2-mutants callus, development is defective [38].

In summary, we identified defects in seed dormancy control, flowering time control, lateral root initiation, and the fate of root hair as well as guard cells which were previously overlooked in H1-depleted plants. This collectively indicates that linker histones are required for a tight control of developmental and cellular transitions.

### H1 variants are necessary to form compact heterochromatin domains but are dispensable for TE silencing and peripheral positioning of chromocenters

**Fig. 2:**
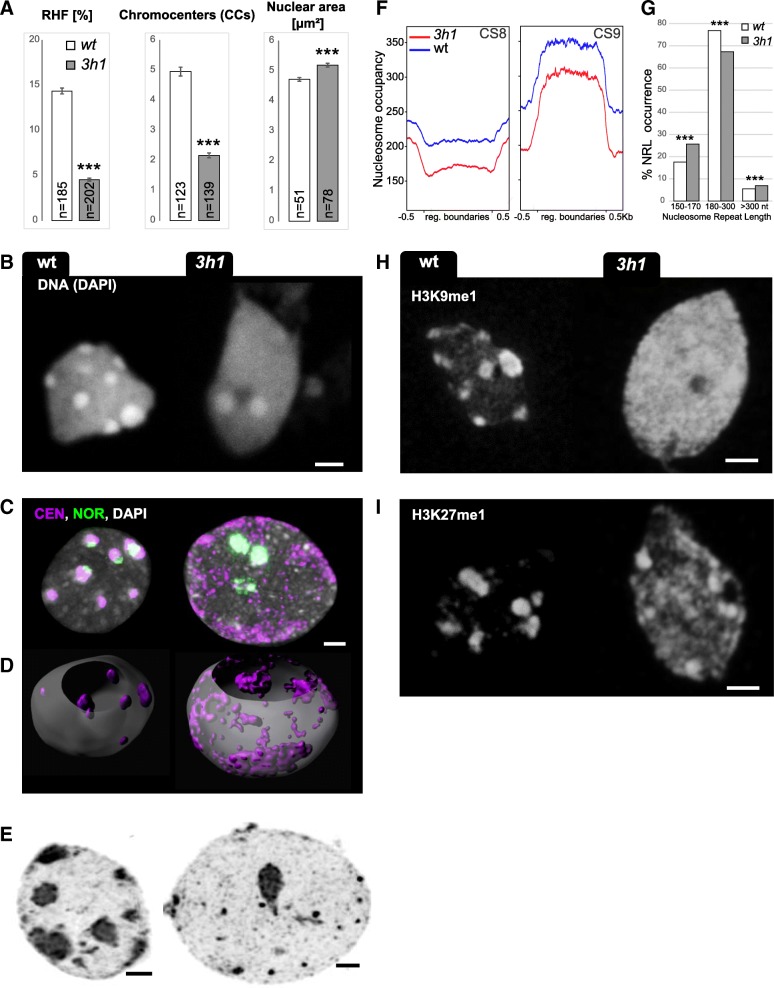
Loss of H1 variants leads to global chromatin decondensation but is dispensable for heterochromatin identity. Cytogenetic (**a**–**e**, **h-i**) and nucleosome profile (**f**, **g**) analyses of chromatin organization in *3h1* triple mutant and wild-type segregant (wt) seedlings. **a** H1 depletion induces a significant reduction of the relative heterochromatin fraction (RHF) and in the number of chromocenters (CCs) as well as an increase in nuclear size (area). ***two-sided *t* test, *p* < 0.001; error bars, standard error to the mean (SEM). Cytological analyses on isolated, spread leaf nuclei: **b** Typical wt and *3h1* nuclei as used in **a**, stained with DAPI. Scale bar, 2 μm. **c** H1 depletion induces a spatial dispersion of the centromeric repeats (CEN, purple) but not of the 45S rDNA, nucleolar organization region repeats (NOR, green) as shown by fluorescence in situ hybridization (FISH). **d** 3D image segmentation of the CEN signals shows that the preferentially peripheral localization of CEN repeats is unaffected in *3h1* nuclei despite their lack of condensation. **e** High-resolution imaging and deconvolution-based reconstruction of *3h1* and wt nuclei. Nanoscopic bodies of condensed chromatin are dispersed throughout the nucleus in *3h1* instead of conspicuous chromocenters as in wt. **f** Nucleosome occupancy is lower in *3h1* heterochromatin, as defined by the chromatin states (CS) 8 and 9 [40]. **g** Distribution of nucleosomal repeat lengths (NRLs) in wt and *3h1*, chi-square test, ***p < 0.0001. **h, i** The heterochromatic marks H3K9me1 and H3K27me1 are not reduced but redistributed in *3h1* nuclei. Scale bar, 2 μm. Isolated leaf nuclei were flow-sorted according to their 2C DNA content (**a**–**e**, **h, i**)

H1.1 and H1.2 variants are largely ubiquitously expressed in plant tissues except in the reproductive lineage [24, 25], while H1.3 is usually not expressed in most cell types [28]. Transient depletion of H1 proteins at the somatic-to-reproductive transition precedes, and probably causes, drastic chromatin changes at the nuclear level [24, 25]. In order to analyze the consequence of H1 depletion on chromatin organization at the cytological scale, we quantified the following parameters in isolated leaf nuclei from *3h1* and compared them to that in wild-type nuclei: number of chromocenters, the relative heterochromatin fraction (RHF) measuring the fraction of chromatin condensed in conspicuous foci or domains [39], and nuclear size informing on the global compaction level of chromatin. These analyses showed that *3h1* nuclei have a stark reduction in heterochromatin content with a RHF of less than 5%, were significantly larger than the wild type and failed to form the typical 6–8 heterochromatic chromocenters (CCs) normally seen in most wild-type somatic nuclei (Fig. 2a, b; Additional file 2: Table S1).

*Arabidopsis* CCs are largely composed of centromeric and pericentromeric transposable element (TE) repeats of the five chromosome pairs, and a subset of two to four CCs comprising ribosomal (rDNA) repeats is associated with the nucleolus [39, 41]. To understand where these repeats were located in the *3h1* nuclei in the absence of conspicuous chromocenters, we carried out 3D fluorescence in situ hybridization (FISH) on intact nuclei embedded in acrylamide gel pads [42]. Centromeric repeats were remarkably dispersed in *3h1*-mutant nuclei, while rDNA repeats were localized in the remaining compact CCs, as in the wild-type nuclei (Fig. 2c). H1 is thus essential for maintaining the structural, compact domains at the (peri-) centromere regions but is dispensable for the heterochromatinization of rDNA repeat loci. Interestingly, the decondensed centromeric repeats in the *3h1* nuclei remain located at the periphery as described in the wild-type nuclei [43] (Fig. 2d). Thus, H1-mediated CC compaction occurs downstream of the spatial positioning of the centromeric regions. In addition, high-resolution imaging indicated the presence of nanoscopic bodies of condensed chromatin in the *3h1* nuclei (Fig. 2e). These were not detected in the wild-type nuclei and therefore could correspond to dispersed heterochromatin regions that were not assembled into chromocenter structures.

The three *Arabidopsis* H1 variants vary in protein structure and expression pattern and may not equally contribute to heterochromatin organization [28]. We quantified heterochromatin content in whole-mount roots and cotyledons in wild-type, mutant, and complemented mutant seedlings. We found an intermediate reduction in heterochromatin content in the double *h1.1h1.2* mutant compared to that in the triple mutant in roots, an effect restored by tagged H1.1 or H1.2 variants (Additional file 1: Figure S4A, B). We explain this by a possible compensatory expression of *H1.3* when H1.1 and H1.2 are depleted, as inferred from reporter analysis in *3h1* roots showing ectopic H1.3-GFP levels (Additional file 1: Figure S4D). However, in cotyledons, which are leaf structures of embryonic origin, CC formation and compaction of centromeric and pericentromeric repeats seem controlled by the H1.1 and H1.2 variants only (Additional file 1: Figure S4C). In wild-type plants, the relative levels of H1.1 and H1.2 variants change along the meristematic-elongation differentiation transition in roots (Additional file 1: Figure S5) suggesting a specialization of these variants, however with a tissue-dependent, functional redundancy revealed in our mutant complementation analyses.

To investigate whether heterochromatin dispersion in *3h1* correlates with looser chromatin organization at the molecular level, we generated chromatin accessibility profiles using micrococcal nuclease sequencing (MNase-seq). We focused the analysis on nucleosomal coverage and distribution in the regions characteristic for heterochromatin and indexed as chromatin states CS8 and CS9 in the established nomenclature [40, 44]. In *Arabidopsis*, CS8 and CS9 are enriched in DNA methylation, H3.1 histone variant, and H3K27me1 and H3K9me2 modifications. In our analyses, the typical CS8 and CS9 regions have a consistent 12–15% reduction in nucleosomal density in the *3h1* nuclei (Fig. 2f, Additional file 1: Figure S6). In addition, nucleosome distribution is more variable in *3h1* heterochromatin as shown by the broader distribution of MNase-protected regions compared to wild type: notably, we found a higher frequency of both short (< 150 nt) and unusually long (> 300 nt) fragments, while the average nucleosome repeat length (NRL) was globally shorter by 10 nt in the *3h1* mutant (Fig. 2g). We concluded from these analyses that H1 constrains nucleosomal spacing and enhances regularity in nucleosome distribution along the heterochromatin regions in *Arabidopsis*. Variability in nucleosomal spacing and distribution in heterochromatic regions might be responsible for the unstable chromocenters in the *3h1* nuclei. Interestingly, the absence of microscopically visible chromocenters does not seem to impair the deposition of their corresponding epigenetic silencing marks which remain abundant and widely redistributed (Fig. 2h-i) most likely following the dispersion of cognate heterochromatin regions.

We then asked whether this structural and spatial dispersion of heterochromatin in *3h1* is associated with reduced silencing of transposable elements (TEs) which are typically confined to chromocenters. Strikingly, RNA-seq profiling of wild-type and *3h1* seedlings did not show changes for the vast majority of TE transcript levels. This demonstrates that H1-mediated heterochromatin condensation into larger compact CCs is dispensable for silencing of the majority of heterochromatic TEs (Additional file 3: Table S2 and Additional file 4: Table S3). Nevertheless, a moderate number of 450 elements (*p* value < 0.05 and fold change > 2) representing about 1.5% of TAIR10-annotated TEs were significantly reactivated in *3h1* plants. A third of them corresponded to LTR/gypsy elements that are mainly distributed within the pericentromeric regions (Additional file 1: Figure S7, Additional file 3: Table S2, Additional file 4: Table S3).

Collectively, our observations indicate that linker histones contribute to regular nucleosomal distribution and density over heterochromatin regions and are absolutely required for the formation and/or maintenance of conspicuous chromocenter domains.

### H1 variants enable a regular spatial distribution of nanoscale chromatin domains and regulate nucleosomal density and mobility in euchromatin

**Fig. 3:**
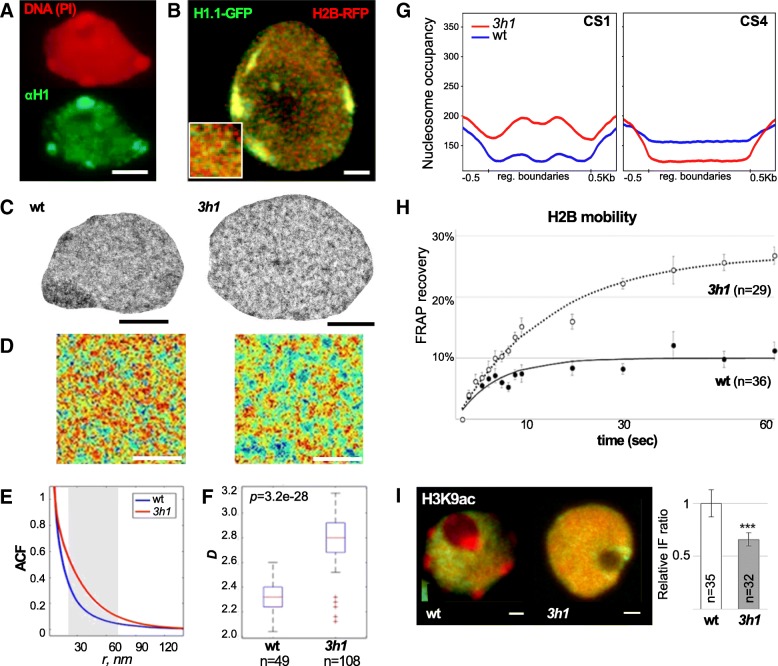
H1 depletion has a strong impact on euchromatin organization with increased dispersion of nanoscopic domains, altered distribution of nucleosome coverage, and increased mobility. **a**, **b** H1 is abundant in euchromatin distributed as discrete foci partially colocalizing with H2B. **a** H1 immunostaining and propidium iodide (PI) counterstaining as in Fig. 1 (fixed, spread nuclei; scale bar, 2 μm). **b** Representative distribution of GFP- and RFP-tagged H1.1 and H2B imaging in the nuclei from fresh root tissue (epidermis, single plane, confocal laser scanning microscopy). The inset shows a close up (1 × 1 μm) in euchromatin displaying H1-enriched regions (dominant green signals) at the nanoscale, but below optical resolution. Scale bar, 1 μm. **c**–**f** Ultrastructural analysis of euchromatin organization in wt vs *3h1*. **c** Typical TEM image of the nuclei stained with uranylacetate on 7-nm cryosection (root epidermis, see the “Methods” section). Scale bar, 1 μm*.* **d** Representative region of interest (ROI) in euchromatin of wt and *3h1* nuclei used for spatial autocorrelation function (ACF) analyses. Scale bar, 500 nm*.* **e**, **f** Spatial chromatin density analyses show decreased regularity in the spatial chromatin distribution pattern in *3h1* revealed by a less shallow ACF curve within length scales of 20–60 nm (gray zone, graph, **e**) and higher dispersion of length scales as shown by a bigger range of the estimate *D* characterizing the spatial autocorrelation fit (**f**). These differences in *3h1* are restored upon complementation with an H1.1 expressing construct. ***Unpaired *t* test, *p* < 0.001, see also Additional file 1: Figure S8. **g** Nucleosome coverage but not qualitative distribution is altered in H1-depleted euchromatin. Antagonist effects are seen for regions of chromatin states CS1, 3, and 7 (CS1 only is shown here) and CS4 (CS according to [40]), see also Additional file 1: Figure S6 for nucleosome occupancy in *3h1* and wt over regions from all chromatin states. **h** H2B-RFP fluorescence recovery is ~ 2.5-fold faster in *3h1* compared to in wild-type as measured in FRAP experiments, see the “Methods” section. **i** Histone acetylation levels are lower in the *3h1* leaf nuclei compared to wt; *t* test, *p* < 0.001, see also Additional file 2: Table S1. Scale bar, 2 μm

As shown by genome-wide profiling, *Arabidopsis* H1 variants are abundant throughout the genome and, besides heterochromatin, are present in the euchromatin regions [28, 29]. Immunolocalization with an antibody specifically targeting plant H1 variants allowed visualizing the native distribution of H1 in situ, revealing discrete regions in euchromatin and encompassing heterochromatic CCs (Fig. 3a). Furthermore, H1.1-GFP was found to be distributed homogenously in euchromatin with, however, the occurrence of enrichment foci relative to nucleosome distribution visualized with an RFP-tagged H2B variant (Fig. 3b). We thus asked whether H1 depletion also impacts the structural organization of euchromatin regions. To resolve nanoscale level of organization, we measured chromatin density patterns on ultrathin transmission electron microscopy (TEM) preparations (Fig. 3c). For this, we used a spatial pattern analysis approach that was previously validated to capture relevant structural features of chromatin organization in cancerogenous animal cells [45] and that we adapted for images of the *Arabidopsis* nuclei [46]. In brief, a spatial autocorrelation function (ACF) of chromatin staining spatial distribution is calculated inside multiple regions of interests (ROIs, Fig. 3d, Additional file 1: Figure S8A) within the euchromatin region of each nucleus and is used to infer the distribution of structured signal intensities at given length scales (Fig. 3e). In wild-type nuclei, the spatial autocorrelation fit (ACF) is shallow, indicating high autocorrelation (i.e., highly regular arrangements) of nanodomains within length scales of ~ 40 nm. By contrast, the exponential decrease of the ACF fit of *3h1* euchromatin was dampen compared to the wild-type nuclei, particularly within 30–60 nm length scales (gray range, graph Fig. 3e). This means that chromatin densities also distribute over large length scales in the mutant. This characteristic is also quantified by measuring the shape of the density ACF (*D* value in box plot Fig. 3e). This showed a higher dispersion of length scales in *3h1* compared to the wild-type nuclei (Fig. 3g). In other words, *3h1* euchromatin shows a significant loss of spatial homogeneity with chromatin nanodomains (high-density patches) spatially distributed along variable intervals (length scales) compared to a highly regular distribution in wild-type chromatin. This trend was reversed in mutants complemented by a tagged H1.1 variant (Additional file 1: Figure S8B) and confirmed in an independent quantification made on fluorescently immunolabeled nucleosomes captured by super resolution microscopy (Additional file 1: Figure S8C).

We then investigated the euchromatin structure at the molecular level and analyzed both nucleosome occupancy profiles and density in our MNase-seq experiments along with defined genomic elements. Interestingly, in *3h1*, the shape of nucleosome occupancy profiles over the most prevalent chromatin state (CS) [40] is comparable to that of the wild-type samples (Additional file 1: Figure S6). However, nucleosomal density is clearly affected in *3h1* chromatin. Comparative density profiles for each chromatin state showed enhanced or diminished average density levels relative to the element boundaries, as for instance, the CS1- and H3K27me3-marked CS4 states, respectively (Fig. 3g, Additional file 1: Figure S6). This is interpreted as a partial loss of structural differentiation of the chromatin states in the absence of H1. As a comparison, we analyzed the impact of the loss-of-function of the CAF-1 histone chaperone contributing nucleosome assembly on nucleosome density distribution across these CS as done for [47]. The analysis showed some alteration in global levels for some but not all CS states but without modifying the profile amplitudes as H1 depletion does (Additional file 1: Figure S6).

Modular alterations in nucleosome density profiles in *3h1* may possibly arise from decreased structural constraints facilitating nucleosome redistribution compared to in wild type. To address the question of whether nucleosome turnover is increased in the absence of linker histone, we carried out fluorescence recovery after photobleaching (FRAP) analyses on the root epidermal nuclei expressing an RFP-tagged H2B reporter [48] in a wild-type or *3h1* background. The analysis showed a ~ 2.5 times faster recovery in H1-depleted chromatin compared to in the wild-type nuclei (Fig. 3h). Specifically, while the diffusional component of the recovery curve (until ~ 10s) appeared similar in both wild-type and mutant chromatins, H2B-RFP recovery rapidly reaches the slow phase characteristic of limited binding-unbinding events in wild-type chromatin [49]. The steep and continuous recovery in *3h1* indicates that H1 depletion unleashed constraints in chromatin organization permitting rapid turnover of H2B-RFP. Furthermore, this chromatin property measured in *3h1*-mutant differentiated root cells resembled that of the property of wild-type chromatin in meristematic cells (pluripotent and undifferentiated) cells (Additional file 1: Figure S9). This increased mobility did not, however, coincide with higher levels of histone acetylation as found in meristematic cells [50]. By contrast, a moderate decrease was measured in immunostaining (Fig. 3i, Additional file 2: Table S1) which was further confirmed by immunoblotting on whole seedling chromatin extracts (Additional file 1: Figure S16A,B).

We concluded that H1-depleted cells harbor a relaxed and highly mobile chromatin with a low degree of structural differentiation between chromatin states. These analyses thus uncover a role for *Arabidopsis* H1 in euchromatin which has not been described previously.

### H1 loss-of-function allows identifying both H1-sensitive and H1-independent genes

**Fig. 4:**
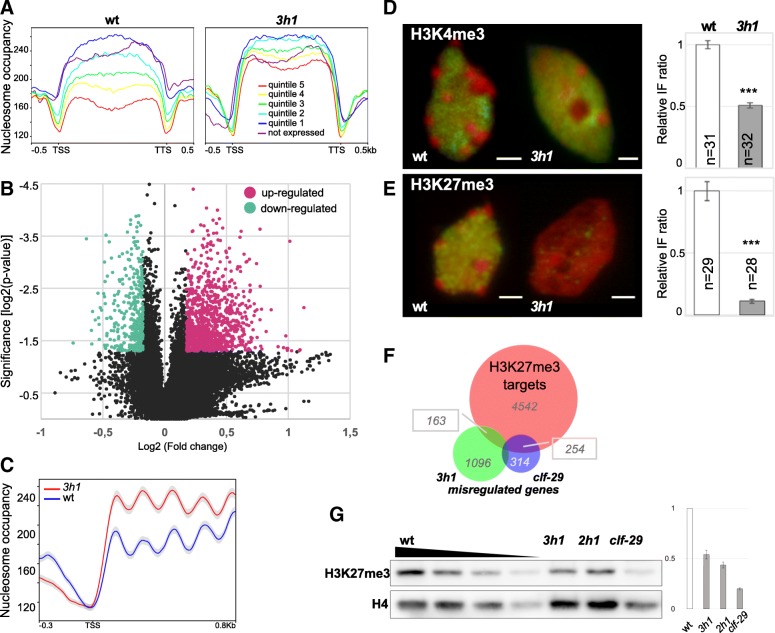
H1 is necessary to secure transcriptional state-specific nucleosomal and epigenetic profiles yet influence only a moderate gene fraction. **a** Nucleosome distribution profiles clearly define distinct gene classes according to expression levels in wild type but no longer in *3h1*. Quintiles 5 to 1 represent categories of genes with expression levels ranked from the highest to the lowest level, respectively, as previously described [28]. **b** H1 depletion induces moderate changes in the transcriptional profile, yet a subset of 701 genes (*p* < 0.05 and fold change > 2) is misregulated. The volcano plot was cropped around the denser part of the dataset. The full plot is presented in Additional file 1: Figure S10. **c** Upregulated loci show a characteristic nucleosome occupancy with high periodicity and a higher coverage in *3h1* downstream the TSS. TSS, transcription start site. **d**, **e** Decreased abundance of H3K27 and H3K4 trimethylation (green, immunosignals (Ab); red, propidium iodide (PI) counterstaining) in *3h1* measured by quantitative immunostaining on isolated leaf nuclei. The graphs shows the relative abundance of these respective histone modifications (Ab/PI ratio relative to wt). Scale bar, 2 μm. **f** Genes which are upregulated (*p* < 0.05) in *3h1* share a significant overlap with H3K27me3 targets defined by [52] (*p* = 0.0007, Fisher exact test) but remain distinct from those affected by the *clf-29* mutation [53]. **g** A twofold reduction of H3K27me3 levels upon H1 depletion is detected by Western blot on chromatin extracts from seedlings yet is less dramatic than in a loss of PRC2 function mutant, *clf-29*. Left panel: the blots show H3K27me3 and total histone H4 levels (loading control) detected on the same membrane. A dilution series of wild-type samples, together with the absence of signal saturation in luminescence imaging, allows showing proportionality of detection signals with protein amounts. The two original gel blots are presented in Additional file 1: Figure S15. Right panel: the histogram shows mean H3K27me3 relative to H4 signals from three individual biological replicates. The wild-type level has been arbitrarily set to 1. *3h1*, triple H1 mutant; *2h1*, *h1.1h1.2* double mutant

Next, we asked whether euchromatin relaxation induced by H1 depletion was reflected at the molecular level. Combined analysis of MNase-seq and RNA-seq profiles allowed us to infer correlations between nucleosome occupancy and relative transcription level grouped into quantiles in wild-type and *3h1* seedlings. As previously reported [51], nucleosomal coverage inversely correlates with gene expression levels in wild-type tissues (Fig. 4a). Low nucleosomal density over highly expressed genes is commonly interpreted as a result of, if not a requirement for, high RNA polymerase accessibility linked with high transcriptional rates. In *3h1*, we observed a notable reduction of the structural differentiation among transcription levels with a general tendency for higher nucleosomal density, specifically downstream of the transcriptional start sites (TSS) (Fig. 4a).

Surprisingly though, this higher nucleosomal occupancy over genes was not correlated with a massive deregulation of transcription. Indeed, comparison of *3h1* transcriptomes determined that about 701 genes are misregulated as compared to wild-type plants (*p* value < 0.05 and fold change > 2, Fig. 4b, Additional file 1: Figure S10, Additional file 5: Table S4). Taken together, these observations have two important implications: (i) H1-mediated nucleosome occupancy is a structural signature correlating with gene expression levels and (ii) for a minor group of ~ 700 H1-sensitive genes, H1-mediated nucleosome occupancy is epistatic to transcriptional regulation. This invites revisiting the idea that nucleosome density influences transcription.

The class of H1-sensitive genes is characterized by 43 down- and 658 upregulated loci in *3h1* seedlings (Fig. 4b, Additional file 5: Table S4, Additional file 1: Figure S10). Analyzing functional categories represented among downregulated genes clearly showed a collective role in light-related metabolism (Additional file 4: Table S5, Additional file 5: Table S4). The group of upregulated genes, however, did not show any specific enrichment in GO terms (not shown) nor a dramatic overrepresentation of specific chromatin states (Additional file 1: Figure S11). Nevertheless, it is noteworthy that they displayed a high periodicity in nucleosome positioning within 800 bp downstream the transcriptional start site (TSS) indicating a strong phasing for this class of genes (Fig. 4c). Highly phased nucleosomes profiles are thought to be a feature of highly expressed genes [54], whereas, intriguingly, this class of H1-sensitive genes is weakly expressed in wild-type plants (Additional file 1: Figure S12).

Collectively, our data indicate that H1 variants provide structural attributes defined by nucleosome density over gene bodies that differentiate transcriptional states with distinct expression levels. In a counterintuitive manner, H1 depletion only affects the expression of a relatively small set of (701) expressed genes. This observation poses questions particularly when considering the strong, genome-wide alterations in nucleosome occupancy and euchromatin mobility in the *3h1* mutant.

### H1 depletion impairs the distribution of H3K27me3 and to a lesser extent of H3K4me3

We previously described that natural eviction of H1 in spore mother cells (SMC) precedes a breadth of global chromatin changes at the structural and epigenetic levels during the somatic-to-reproductive transition [25]. These include heterochromatin decondensation, histone hyperacetylation, elevation of H3K4me3, and decrease of H3K27me3 levels [25]. These changes are further associated with a transient decrease of DNA methylation levels in the CHH but not the CG sequence context [23]. To assess whether H1 depletion is functionally linked to such chromatin changes, we introgressed the mCG and mCHH DynaMET reporters [23] into the triple *3h1* mutant to analyze the cytological distribution and abundance of DNA methylation. Albeit cytological imaging would not capture locus-specific alterations as those described previously in H1-depleted tissues by molecular profiling [22, 32], the global levels and distribution patterns of methylated DNA in *3h1*-mutant root nuclei were similar to that in wild-type nuclei (Additional file 1: Figure S13). We then performed quantitative immunostaining for the canonical marks H3K4me3 and H3K27me3 previously found to be dynamically redistributed in H1-depleted SMCs [25]. We found that H3K4me3 levels were moderately but reproducibly lower in the *3h1* nuclei compared to in the wild-type nuclei (Fig. 4d, Additional file 2: Table S1, Additional file 1: Figure S14). More dramatically, H3K27me3 levels were considerably lowered in H1-depleted nuclei compared to in wild-type nuclei (Fig. 4e, Additional file 2: Table S1) while H3K27me2 was visibly unaffected (Additional file 1: Figure S14). A two-fold reduction of H3K27me3 and H3K4me3 global levels were further confirmed by immunoblotting on whole seedling chromatin extracts (Fig. 4g, Additional file 1: Figure S15 and Figure S16C,D). Of note, expression of genes encoding PRC2 subunits were not significantly altered in *3h1* plants (Additional file 4: Table S6). Consequently, our observations indicate that linker histones are required for targeting, maintenance, or both, of H3K27me3. This raised the possibility that gene upregulation in *3h1* may indirectly result from downstream consequences of a modified epigenome landscape. Yet only 10% of the upregulated genes overlapped with known H3K27me3 genomic targets. Among those, a small share (4%) corresponds to genes misregulated in the PRC2 mutant *clf* (*curlyleaf*), compromised in histone H3-Lys27 methyltransferase activity [53] (Fig. 4f). Thus, loss of H3K27me3 does not appear to represent a major cause of gene upregulation in *3h1* seedlings. This indicates that altered H3K4me3 and H3K27me3 landscapes may rather be a consequence of altered chromatin structure affecting targeting or spreading of histone modifications, or both.

## Discussion

**Fig. 5:**
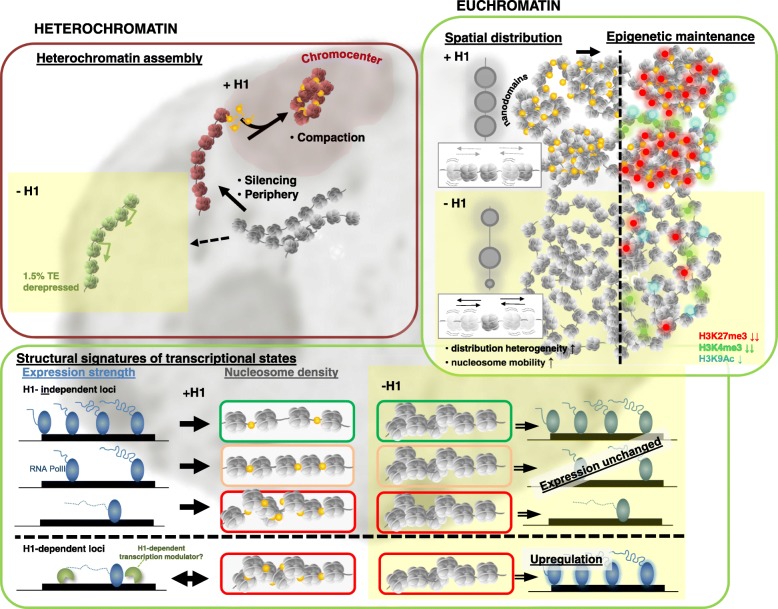
Model for H1 function in heterochromatin and euchromatin organization at the topological and molecular level. Graphical representation of H1 roles on chromatin organization at the cytological (spatial) and molecular level based on analyses reported in this study. Heterochromatin: H1 is dispensable for silencing and peripheral positioning of the vast majority of heterochromatic repeats but necessary for their condensation into compact chromocenter domains; yet a subset of transposable elements is directly affected by H1 and become derepressed in its absence (yellow box, -H1). This indicates both H1-independant and H1-dependent TE silencing controls. Euchromatin: top right panel, H1 is necessary to provide homogeneity in chromatin topology and spatial organization of chromatin domains. H1 depletion results in both larger gaps between nanodomains, possibly enabling increased accessibility, and irregular, high local compaction; this chromatin heterogeneity is reminiscent of H1-depleted pluripotent cells [9], cells with a loss of a SWI/SNF chromatin remodel function or undergoing tumorigenic reprogramming [57]. Concomitantly, H1-depleted chromatin displays increased mobility and poor maintenance of histone H3 lysine 4 (green) and more strongly lysine 27 (red) methylation. At the molecular level (lower panel), H1 provides distinct structural signatures (nucleosome coverage) at loci marked by distinct expression rates but is not epistatic to transcriptional control for a majority of them (H1-independent regulation); a subset of genes (ca 600 under a stringent cutoff), however, displays an H1-dependent control possibly involving transcriptional regulators directly influenced by H1

H1, due to its inherent properties in promoting a higher order of chromatin folding, has long been considered a typical structural chromatin protein [55]. Accordingly in plants, H1 was shown to induce ectopic heterochromatinization when expressed in a heterologous system [56]. In addition, like in animals, plant H1 variants interplay with the DNA methylation machinery, particularly affecting TEs. H1 also affects DNA methylation at the genic regions, although only a few have been experimentally interrogated so far [22, 31–33]. Despite their expected fundamental role in chromatin accessibility, very little was known about the specific impact of H1 at the structural, epigenetic, and gene expression level in plants. In this study, we unveiled distinct effects of H1 function on nucleosome occupancy and transcriptional activity over heterochromatin and protein-coding genes, respectively (model presented in Fig. 5). We further unveiled that H1 is strictly required for heterochromatin formation but not for the establishment of heterochromatic hallmarks, that H1 provides spatial regularity in nanodomain chromatin compaction and distribution which coincides with a role in the maintenance of H3K27me3 in euchromatin, and that *in fine* H1-mediated chromatin organization secures a proper control of specific developmental transitions.

### H1 as a heterochromatin architect largely uncoupled from transcriptional silencing

We showed that H1 plays an unexpected role in heterochromatin regulation in *Arabidopsis*. This function is largely but not entirely uncoupled from transcriptional silencing, as shown by massive relaxation of peripheral heterochromatin and minimal reactivation of TE expression in plants lacking H1. H1-dependent chromocenter formation also appears to occur downstream the epigenetic marking of heterochromatin, notably DNA, H3K9, and H3K27 methylation, which remain abundant in *3h1* nuclei. Consistently, heterochromatin dispersion despite strong H3K9me2 marking was also reported in *2h1* leaf nuclei (i.e., lacking H1.1 and H1.2 variants) in a study published during our manuscript revision [58]. In the same study, the absence of H1 in the vegetative cell of pollen grains correlates with the activation of about, and only, hundred TEs [58]. Collectively, these observations argue against the common view that heterochromatin compaction in chromocenter domains is directly responsible for the general transcriptional repression of TEs. Transposable elements upregulated in *3h1* define a functional category of loci where H1-mediated chromatin organization primes over other controls of transcriptional silencing. Those TEs are mostly pericentromeric and enriched in LINE, gypsy, and copia elements. DNA methylation at pericentromeric TEs is independent of the RNA-dependent DNA methylation (RdDM) pathway but requires the DNA methyltransferase CHROMOMETHYLTRANSFERASE2 (CMT2) and the chromatin SWI/SNF remodeler DECREASE in DNA METHYLATION1 (DDM1) [32]. At pericentromeric TE loci, H1 is thought to modulate (but not hinder) DNA methylation in the CHG context by reducing access to CMT2, a configuration resolved by DDM1 [32]. This explains the global gain of pericentromeric TE CHG methylation in the absence of H1 [32]. This global effect does not explain the derepression of H1-sensitive TEs. The observation that H1 can interact with CMT3 [59] suggests also a scenario in which H1-bound nucleosomes are favored for CMT3-mediated DNA methylation, thereby reinforcing silencing at these TE loci. Thus in *Arabidopsis*, H1 may play a dual role in restricting methylation through antagonizing DDM1 function [32] but also reinforcing methylation in a CMT3-dependent manner. Such a model, however, remains to be experimentally validated. Alternatively, and possibly not exclusively to other mechanisms, the absence of H1 may expose additional TEs to the DNA demethylase DEMETER; this was suggested for TE loci activated in the vegetative cells of pollen grains where the main H1 variants are naturally depleted [58]. In animals, interestingly, H1 depletion also leads to partial TE reactivation but following distinct mechanisms: independently of common epigenetic silencing marks in mammalian stem cells [60] or through an H3K9me2-dependant manner in *Drosophila* [14, 61].

Chromocenters containing ribosomal DNA repeats are not affected by the loss of H1 variants in *Arabidopsis*, further indicating the existence of an H1-independent control of such heterochromatic structures. It would be interesting to investigate whether other structural proteins such as H1-related, GH1-containing proteins [21] contribute NOR condensation or if hypoacetylation of the NOR nucleosomes by HDA6 [62] is self-sufficient.

In addition, dispersed heterochromatin regions still locate peripherally in the *3h1* nuclei indicating that not only structural compaction but also peripheral location is uncoupled from heterochromatin silencing in *Arabidopsis*. Interestingly, this role in chromocenter formation is not a shared, inherent feature of linker histones among eukaryotes. Indeed, depletion of the H1c and H1d mammalian variants in mouse embryonic stem cells leads to chromocenter clustering but not decondensation [60]. In *Drosophila*, H1 also promotes the condensation of pericentromeric heterochromatin, but this observation is specific to salivary gland cells which are singular due to their polytenic chromosomes [20]. In both *Drosophila* and mouse somatic cells, chromocenter condensation seems instead to be controlled by the D1 and HMGA1 multiAT hook proteins, respectively [63]. Therefore, our study uncovers an influence of H1 in chromocenter condensation which may differ from the animal system.

### H1 as a fine-scale architect of euchromatin organization from nucleosomes to nanodomains

We have shown that global chromatin decondensation in H1-depleted nuclei correlates with a greater heterogeneity in the spatial distribution of chromatin domains in the nucleoplasm. Yet, compact nanodomains as those visualized by high- and ultrastructural microscopy imaging still form in the absence of H1. This is consistent with the intrinsic property of chromatin fibers to fold along various configurations even in the absence of linker histones: in vitro assembly assays have shown that nucleosomal arrays can be formed in the absence of H1, yet with a lesser degree of molecular organization, forming “ladder” or “puddle” type of arrangements [64]. Thus, our observations in vivo might relate to this intrinsic property of H1. Furthermore, the increased spatial dispersion of compact nanoscopic domains free of H1 in *3h1* nuclei is highly reminiscent of euchromatin organization in tumorigenic nuclei losing fractal property of organization [45].

At the molecular level, we observed that *Arabidopsis* H1 influences nucleosomal spacing, resulting in deregulated density over defined genomic regions. Notably, H1 depletion affects the structural differentiation, in terms of nucleosomal density, of chromatin states. This effect is distinct from that of a loss-of-function of a general chromatin factor regulating nucleosome assembly such as the histone chaperone CAF-1 (Additional file 1: Figure S6). In addition and strikingly, besides a shift of NRL distribution peak from 170–180 to 160–170 bp similar to the situation in animals [10], *3h1* chromatin harbors a higher fraction of both short NRLs (< 160 bp) or unexpectedly long (> 200 bp). This indicates a relaxed control of nucleosome distribution, with a more permissive (i.e. less constraint) nucleosome positioning [64]. H1 incorporation is thought to reduce the sterical occupation of linker DNA and to favor a compact zig-zag chromatin folding [65]. The question arises, how NRL longer than 197 bp can be achieved. One possibility is that such NRL corresponds to the MNase inaccessible regions spanning two closely associated nucleosomes; another possibility is suggested by the occurrence of the so-called stretched nucleosomes where DNA-histone contacts are lost in the absence of H1 [66]. Ultimately, variations in NRL and associated linker DNA length can contribute to chromatin fiber polymorphisms with in turn an influence on the formation of chromatin domains [67–69]. The relaxed NRL distribution hence variable nucleosome positioning landscape seen in *3h1* is thus likely responsible for the observed local variations in chromatin compaction and spatial heterogeneity.

How this fine-scale topology connects with chromatin function, notably gene expression, requires a biophysical appraisal of chromatin organization. Emerging models linking molecular-to-spatial (macromolecular) levels of chromatin dynamics are very attractive to explain the emergence of transcriptional dynamics during cellular differentiation [70]. Notably, local nucleosomal arrays appear to connect with molecular crowding, a dynamic property directly influencing the access and residence of the transcription machinery to chromosomal regions [57, 70]. In such a model, spatial heterogeneity of chromatin nanodomains seems to favor the occurrence of transcriptional bursts such as those observed in an animal model of cellular differentiation [57, 70]. In addition, specific disturbances in spatial chromatin organization in cancerogenous cells have been correlated with transcriptional heterogeneity linked to the paired effect of increased accessibility and increased local compaction [57]. It is tempting to link these biophysical, molecular models of the nanoscale level of chromatin organization with the role of linker histones. Consistent with it, pluripotent mammalian cells in which H1 levels are low display highly dispersed, small nucleosome clutches favoring RNA PolII redistribution [9]. Collectively, in light of these models and observations, we propose that the molecular and spatial distribution heterogeneity of H1-depleted chromatin in *Arabidopsis* resembles that of animal un-/de-differentiated cells. This proposal invites future studies on the role of *Arabidopsis* H1 variants in regulating transcriptional dynamics notably during cellular differentiation or in response to environmental cues.

### H1 defines a dual causality between chromatin organization and gene expression

Our cytological analyses clearly showed a profound impact of H1 on hetero- and euchromatin organizations at the molecular and spatial distribution level. But the daunting question remains whether this influences chromatin function and notably gene expression. Our study uncovers an unexpected, complex relationship between chromatin organization, probed by nucleosomal coverage, and gene expression. For instance, we found a lower nucleosome coverage in *3h*1 along a large fraction of genes (excluding heterochromatic regions, discussed earlier), yet this reduced nucleosome occupancy did not trigger a significant upregulation in *3h1* plants. This was true for the genomic regions associated with H3K27me3 (e.g., chromatin state CS4, [40]), hence PRC2-target loci. By contrast, the genomic regions normally associated with transcriptional competence (e.g., CS1 enriched in H3K4me3 and H3.3) gained nucleosomal coverage in *3h1*, still without detectable consequences on most transcript levels. Thus, for a large fraction of the genome, both in eu- and heterochromatin contexts, H1-mediated chromatin organization appears to act downstream of the transcriptional controls. The observation that the structural differentiation of loci relative to the gene expression strength is blurred in *3h1* reinforces the conclusion that gene body nucleosome density is not epistatic to transcription.

These findings challenge a common view that transcription is influenced by nucleosome density, coming from the observation that nucleosome occupancy is inversely correlated to gene expression level ([51] and this study). Whether H1-mediated chromatin organization serves another purpose, for instance, facilitating the rewiring of transcriptional programs when cells are environmentally challenged or securing transcriptional robustness is an exciting possibility to be investigated. If they hold true, these concepts would provide an explanation for the developmental phenotypes in *3h1* plants with an apparent relaxed control of several developmental transitions. These deficiencies can be interpreted as instable transcriptional states releasing seed dormancy, flowering, lateral root organ initiation, root hair, and stomatal cell fate earlier than in the wild-type counterparts, although the severity of such developmental phenotypes might be attenuated by the buffering capacity of developmental processes inherent to plants [71].

The dual role of H1 is unveiled by the several hundred of H1-sensitive loci for which chromatin organization has a prime contribution with respect to transcriptional control. Gene upregulation in *3h1* correlated with a notable nucleosome enrichment in the gene body but not at the upstream elements. Yet, whether this average increased coverage effectively associates with high transcriptional rates or whether it is contributed by a fraction of cells harboring silent loci cannot be resolved in bulk tissue profiling. Possibly for these loci, increased nucleosome mobility provides a chromatin template favorable for transcription and/or unfavorable to propagate repressive epigenetic marks, as suggested by compromised H3K27me3 maintenance in *3h1*.

### A new connection between H1 and epigenetic reprogramming

To a certain extent, chromatin organization and properties in *Arabidopsis 3h1* mutant nuclei are reminiscent of a pluripotent chromatin state described in different plant cell types and studies: (i) chromatin decondensation and reduced H3K27me3 abundance resemble the chromatin status of H1-depleted spore mother cells (SMC) at the somatic-to-reproductive fate transition [25, 72]; (ii) heterochromatin decondensation and redistribution of epigenetic marks such as those observed in H1-depleted nuclei are hallmarks of in vitro de-differentiated cells and at flowering transition [73, 74]; and (iii) high chromatin mobility in H1-depleted chromatin is reminiscent of the chromatin state in meristematic cells [50].

These collective observations allow to propose the hypothesis that H1 depletion contributes to reprogramming of the chromatin landscape at the structural and epigenetic level in plants cells undergoing fate reorientation as in the cases mentioned above. In support of this hypothesis, which of course calls for further investigations, the stark decrease in H3K27me3 levels in H1-depleted chromatin such as in *3h1* plant cells or in wild-type SMC [25, 72]. H3K27me3 is notoriously associated with the memory of epigenetic states and contributes gene expression reprogramming during cell pluripotency acquisition and differentiation in animal and plants [38, 75–78]. Interestingly, H1 depletion in mammalian cells, while moderately affecting global transcriptome patterns as here in *Arabidopsis*, affects more particularly pluripotency genes [79].

In addition, and consistently with our proposal, the developmental phenotypes observed in *3h1*—including delayed release of seed dormancy, precocious flowering, deregulated lateral root and root hair initiations, and the control in stomatal spacing—are reminiscent of phenotypes arising in PRC2 loss-of-function mutants with altered H3K27me3 levels [38, 80–85].

The influence of H1-mediated chromatin structure on H3K27me3 levels is a conserved feature in plants (as shown by our study) and in animals [10] although this may be cell type-dependent [13]. An explanation for this may be a substrate preference of the PRC2 complex to H1-containing nucleosomes as shown for the animal EZH2 subunit in vitro [86] leading presumably to a more efficient propagation of H3K27me3 in chromatin states structured by H1 in vivo. Conversely, H1-depleted cells may inefficiently propagate H3K27me3 landscapes, which over numerous divisions may lead to drastic loss, as measured in (terminally differentiated) plant tissues (this study) and in mouse tissues [10]. Involving H1 in H3K27me3 maintenance rather than the establishment is a hypothesis supported by the observation that H1-depleted plants are not impaired in basic growth, nor organogenesis or body patterning processes, but, instead, show altered controls of developmental and cellular transitions normally modulated by PRC2.

## Conclusions

This work explored for the first time the functional relationship between fine-scale chromatin organization at the molecular and spatial levels, transcriptional control (Fig. 5), and phenotypic impact in *Arabidopsis*. Our analyses of the impact of H1 depletion in *Arabidopsis* unveil a remarkable evolutionary convergence of H1 function in plant and animal kingdoms despite a diversification of the H1 variants family [87]. In *Drosophila* and mammalian cells, H1 depletion generates large-scale chromatin alteration including blurring of topological domains, relaxation of nucleosome distribution periodicity, and impaired propagation of the epigenetic marks [10, 88–90]. In *Arabidopsis*, the misregulation of a small proportion of genes in the absence of H1 is intringuing when considering the massive structural chromatin alterations. While it suggests conserved functional principles for H1, it also unveils a dual, causal relationship between transcriptional controls and H1-mediated chromatin organisation. Our findings prompt revisiting the common view that chromatin compaction and nucleosome density influence gene expression in a major way. This idea may only hold true for a subset of H1-sensitive genes and TEs. But generally, transcriptional regulation remains epistatic to H1-mediated chromatin organization for a majority of heterochromatin and euchromatin loci. Based on these findings, we propose that chromatin organization in the absence of H1 loses versatility and is less prone to rapid reprogramming at the structural, epigenetic and transcriptional level. This property is not challenged under controlled growth conditions hence requires testing in modular and variable environments. We suggest a working model, in which linker histones act as fine-scale chromatin architects with a dual function in (i) the facilitation of transcriptional reprogramming (in development or under environmental cues) and (ii) securing stable transcriptional landscapes buffering against variability. Our findings echo with recent models in mammalian cells implicating higher-order folding chromatin topology as an independent route influencing transcriptional dynamics [57] and in facilitating transcriptional robustness in differentiation [70].

## Methods

### Plant materials and growth conditions

The *Arabidopsis thaliana* plants used in all experiments were in the Col-0 background unless it is specified otherwise. The *h1.1h1.2h1.3* (*3h1*) mutant was described before [25, 28]. The snapshots from RNAseq reads of H1 variant expressions in *3h1* and wt are presented in Additional file 1: Figure S17. The mutant showed no detectable levels of H1 in immunostaining experiments [25] and in Western blot (www.agrisera.com/en/artiklar/h1-histone-h1.html). Complemented mutant lines were generated by transforming *3h1* via floral dip method [91] with H1-tagged variants (prom. H1.1::H1.1-RFP, prom. H1.2::H1.1-(G/C) FP, prom. H1.3::H1.3-GFP) described previously [25, 28]. The *3h1* was complemented with either two main (H1.1, H1.2) or all three H1 variants to generate the following lines: *3h1-comp*^*1,2*^*= h1.1h1.2h1.3;H1.1-RFP;H1.2-GFP* (line #KR276), *3h1-comp*^*1,2,3*^ = *h1.1h1.2h1.3;prom. H1.1::H1.1-RFP;prom. H1.2::H1.2-CFP*;prom. H1.3::H1.3-GFP (lines #KR264 and #KR265). For FRAP experiments, the UBQ10::H2B-RFP [48] was crossed with *3h1*, and in the subsequent generations, by genotyping the *3h1*/UBQ10::H2B-RFP and wt segregants were identified.

Seeds were surface sterilized and rinsed in sterile water before transferring onto germination medium (0.5× MS medium, 0.8% agar). They were placed on the medium using toothpicks to ensure a uniform distribution, stratified 2–4 days at 4 °C, and transferred into a plant growth incubator (Percival, Germany) with long-day photoperiod (16 h, 22 °C day/8 h, 18 °C night) and light flux around 120 μM s^−1^ m^−2^ for routine experiments. Growth of calli and scoring of lateral root production were tested under continuous light (light flux around 100 μM s^−1^ m^−2^, Aralab FitoClima 1200). When the flowering stage was necessary, the 10-day-old seedlings were transferred into the soil and grown at 19–21 °C with a 16-h day/8-h night photoperiod.

### Chromatin analyses and immunostaining

The nuclei area, heterochromatin (RHF, CCs), and immunostaining analyses were carried out essentially as described [92] with minor modifications. The nuclei were isolated from rosette leaves of 3–4-week-old seedlings; per extraction, five leaves were fixed during 20 min under vacuum in a fresh 4% formaldehyde solution prior to isolation and resuspension of the nuclei in a final volume of 1 mL nuclei isolation buffer (NIB). DAPI was added at a concentration of 0.1 mg/mL for flow sorting according to DNA content. Diploid (2C) nuclei have been flow-sorted using a BD FACSAria IIIu flow cytometer with a 450/50-nm filter (405 nm laser), equipped with a 100-μm nozzle and 25 Psi pressure. The nuclei were collected in 200 μL of NIB before spreading on Superfrost plus slides (1000 nuclei per slide) and stored at 4 °C until use. Mutant and wild-type plants were grown and processed for nuclei isolation and immunostaining in parallel.

For heterochromatin analysis, the slides were rinsed in SSC2X then PBS before staining with DAPI 1 μg/mL in Vectashield (Vector Laboratory). For immunostaining, the protocol essentially followed previously described steps [92]. As primary antibodies, rabbit anti-Histone H3 (Abcam; ab1791), anti-Histone H1 (Agrisera; as111801), anti-H3K27me3 (Active Motif; 39155), anti-H3K27me1 (Abcam; ab113671), antiH3K4me3 (Abcam; ab8580), anti-H3K9ac (Abcam; ab10812), and anti-H3K9me1 (Abcam8896) were used at a dilution of 1:200 and incubated at 37 °C for 1 h. As secondary antibody, Alexa Fluor 488-conjugated goat anti-rabbit IgG (Molecular Probes; A-11008) was used at a dilution of 1:1000 and incubated for 2 h at 37 °C. The nuclei were counterstained for DNA with propidium iodide (PI).

Sixteen-bit images were acquired using a Leica TCS SP5 confocal laser scanning microscope (CLSM) (Leica Microsystems, GmBH, Germany) using a × 63 GLY lens (NA 1.4) for heterochromatin and immunostaining analyses. Exposure times, illumination intensities, zoom factor, scanning speed, and pinhole were kept identical for the image series in an experiment. For RHF measurements, signal intensities were recorded in manually drawn ROIs capturing chromocenters and normalized over the whole nucleus intensity using Fiji [93]. For immunostaining, the signal intensities for antibodies were normalized against PI levels. Graphs were plotted in Excel, and the data were statistically assessed using a Student *t* test (unpaired, unequal variance) for comparing wild-type and mutant samples.

### Fluorescence in situ hybridization and 3D image processing

#### FISH analysis of leaf nuclei

The nuclei were isolated from leaves of 35-day-old rosettes grown under a 16-h/8-h photoperiod. Nuclei extraction and embedding in acrylamide gel pads on the slide were done as described [42]. Centromeric and 45S rDNA repeats were detected by FISH using pAL1 and pTA9 to generate DNA probes, respectively [39]. FISH was done as described [42] with the following labeling kits and fluorescent immunolabeling reagents: DIG-Nick (Sigma Aldrich, 11745816910), mouse IgG anti-DIG (1:250, Sigma Aldrich, 11333062910), goat IgG anti-mouse IgG~Alexa 488 (1:200, Life Technologies, A11001); Biotin-Nick translation kit (Sigma Aldrich, 11745824910), Biotinylated Anti-Avidin D (1:250, Vector Labs, BA-0300), and Texas Red Avidin D (1:1000, Vector Labs, A-2006). The nuclei were counterstained for DNA with DAPI in Vectashield (Vector Laboratory). FISH signals in 3D nuclei were imaged using Stimulated emission depletion (STED) microscopy (Leica SP8R WL 3xSTED, Leica microsystems, Germany).

#### FISH analysis of cotyledon nuclei

The nuclei were isolated from dissected cotyledons of 5-day-old seedlings grown under a 16-h/8-h photoperiod. Nuclei extraction, fixation, and hybridization with pAL1-derived and F28D6-derived (180-bprepeats) probes [39] were performed as previously described [94]. The slides were washed and mounted in Vectashield with 2 μg/μL DAPI, and image acquisition was performed as in [94].

### Nuclei isolation for MNase-seq

The nuclei were isolated from 3-week-old seedlings frozen in liquid nitrogen as previously described [95] with the following modifications: after resuspending in HBB, the nuclei were applied to a layer of HBB with 40% Percoll (GE Healthcare); centrifuged at 1000*g*, 6 min; resuspended in HBB; applied to 40/75% Percoll gradient; centrifuged at 400*g*, 40 min; collected; and washed three times with HBC. The integrity of the extracted nuclei was monitored using DAPI staining and fluorescence microscopy. The quantity of nuclei was measured by qPCR with primers targeting nuclear DNA.

Digestion was performed by incubating the nuclei suspended in DB buffer (16 mM Tris-HCl pH = 7.6, 50 mM NaCl, 2.5 mM CaCl_2_, 0.01 mM PMSF, 1× Complete EDTA-free Protease Inhibitors (Roche)) with 1.5 μL (final concentration 0.3 U/μL) of micrococcal nuclease (Thermo Fisher), and 2 μL (final concentration 0.2 U/μL) of RNase A (Thermo Fisher) at 8 °C for 90 min with gentle mixing. The reaction was stopped by adding an equal volume of 2× Lysis buffer with EDTA (100 mM Tris-HCl pH = 8, 200 mM NaCl, 50 mM EDTA, 1% SDS). The samples were lysed by incubation at 37 °C for 60 min with shaking (1000 rpm). DNA was purified using phenol-chloroform extraction, precipitated with isopropanol and sodium acetate, and resuspended in water.

DNA was size selected by electrophoresis on 2% agarose gel with 1× TAE buffer with SYBR Gold (Invitrogen) stain. The mononucleosomal band was excised, frozen, and squeezed by 3 cycles of spinning and rehydration on centrifuge column. DNA was purified and concentrated using Agencourt AMPure XP beads (Beckman Coulter). Barcoded libraries were synthesized from 100 ng of mononucleosomal DNA using Ion Xpress™ Plus gDNA Fragment Library Preparation Kit and Ion Xpress™ Barcode Adapters. DNA was end-repaired prior to adapter ligation and size selection, and amplification steps were omitted. The resulting libraries were quantified with Ion Library Quantitation Kit, pooled, and used to prepare the template by clonal PCR with Ion PI™ Template OT2 200 Kit v3 on Ion OneTouch™ 2 System. Sequencing was performed on Ion PI™ chip v2 and Ion Proton™ sequencer using Ion PI™ Sequencing 200 Kit v2 (all Ion Torrent kits and software are trademarks of Thermo Fisher).

### FRAP imaging and data analyses

A *promUBQ10::H2B-RFP* marker [96] was introgressed in *3h1*-mutant plants by crossing. Both wild-type and triple-mutant segregants were analyzed. Measurements were done on the root tips of 2-week-old seedlings grown as previously described. One sample was prepared at a time: the root was excised and delicately mounted (i.e., without squashing) in 0.5× MS between the slide and coverslip (precleaned with EtOH), sealed with transparent nail polish, and let to equilibrate upside down for 10 min on the microscope platform before measurements. The imaging chamber was set at a constant temperature of 20 °C (higher/fluctuating temperatures induce nuclei juggling). Bleaching and imaging were done using an APO PL × 40 oil immersion objective, NA 1.3, over a single plane capturing an optical section of ~ 2 μm encompassing a single nucleus (pinhole opening to 5 AU) with a 256 × 256 pixels image format, threefold zoom factor. Bleaching was performed in euchromatin within ROI of 1 μm diameter using five or more pulses until near-total bleach was obtained (Argon laser at 80% power, 100% transmission in 488 nm), and post-bleach images were recorded using 5–7% laser transmission for excitation, in a series of 10 time points, 1-s interval, followed by 10 time points, 60-s interval. For analyzing the fluorescence recovery, images were first corrected for nuclear drifts occurring during acquisition, using a rigid registration approach in Fiji ([93], plugin/registration/stack reg/rigid transformation). When a single image captured several nuclei, single nuclei were cropped for registration and analysis. Fluorescence measurements were done on the bleach ROI, a control ROI near and outside the nucleus, and over the whole nucleus. Calculation of fluorescence recovery was done as described in [50, 97] whereby the initial intensity was normalized at 1 for each image before average calculation.

### TEM sample preparation, imaging, and image analysis

Seventy-nanometer tissue sections were prepared from 2-week-old seedling roots, using a high-pressure freezing/freeze substitution and uranyl acetate staining approach as described in details previously [46]. The sections from the elongation zone were selected for the analysis (i.e., meristematic zone was avoided), and nuclei pictures were consistently recorded from the epidermal layer at the 24,500-fold magnification yielding a resolution of 1 pixel = 2 nm in our setup. For the analysis, the square regions of interests (ROIs) of similar size (ca 800 × 800 ± 200 pixels) were captured in the euchromatin regions (i.e., excluding strongly staining chromocenters) for the analysis. We used a spatial pattern analysis approach that was previously validated to capture relevant structural features of chromatin organization in cancerogenous animal cells [45]. Spatial autocorrelation analysis delivers a mathematical model of chromatin density distribution for each ROI with respect to the physical length scales within which signal patterns (i.e., local objects of similar intensities) are repeated in a regular pattern (periodicity) [45]. We used a user-friendly graphical interface developed in Matlab for batch processing of multiple ROIs available at https://github.com/barouxlab/ChromDensityNano and described in details previously [46].

### Analysis of developmental transitions

#### Flowering time

Plants for flowering experiments were grown in the greenhouse or growth chamber under the long daylight regime. To avoid positional effect, different genotypes were always randomly arranged over the growth area. The number of rosette leaves was counted when the inflorescence was about 0.5 cm long. Three replicate experiments were conducted: replicate 1 was based on 40 to 50 plants depending on genotype as detailed in Additional file 1: Figure S1A; replicates 2 and 3 were based on 10 and 12 plants per genotype, respectively, as detailed in Additional file 1: Figure S1C,D.

#### Root length and lateral root scoring

Seedlings were grown vertically on square Petri dishes under a continuous light regime. The plates were scanned 8 days after germination to score for the number of lateral roots. Root (main and lateral) lengths were scored using manual vector tracing in Fiji, reported at scale [93]. For microscopic observations of lateral root primordia, 5-day-old seedlings grown under continuous light were fixed in 70% ethanol, rinsed once in sterile water, and mounted in water on microscope slides (five roots aligned/slide covered with 40 × 22 mm coverglass). Primordia were scored according to published developmental scale [98]. Graphs were plotted in R.

#### Root hair observations

Increased root hair number was visible under growth conditions as described above for lateral root scoring. The phenotype was however increased using MS medium supplemented with 5% sucrose, and this medium was used to take the micrographs in Fig. 1.

#### Stomata patterning

Fresh epidermal peals of 14-day-old cotyledons were mounted in water. Images of the adaxial surface were recorded with DIC microscopy, and stomatal clusters were scored following as described [99].

#### Seed dormancy

The experiment was designed as described previously [100] with minor modifications. Plants were grown in a growth chamber under long daylight regime with controlled humidity. Freshly harvested seeds were collected and stored under constant conditions. Around 180 seeds per plant were placed on a wet filter paper in a Petri dish and incubated in the growth incubator at 22 °C under long day light regime. After 3 days, the number of emerging radicles was counted. For the time point “day 1,” the seeds were used 1 day after harvesting. For the time point “3 weeks,” the seeds from the same batch were used 3 weeks after harvesting.

#### Callus induction

Cotyledons from 7-day-old seedlings grown under a 16-h/8-h photoperiod were excised, transferred onto callus induction medium (CIM, Gamborg B5, 0.05% MES, 2% glucose, 0.1 mg/L kinetin, 0.5 mg/L 2,4-D), and let to develop for 5 weeks under a 16-h/8-h photoperiod. Callus size (area) was determined from images using manually drawn contours in Fiji [93]. Graphs were plotted in R.

### Immunoblot analyses

Seeds were surface sterilized in 70% ethanol 0.05% SDS for 3 min and rinsed with 90% ethanol before drying and plating on MS medium supplemented with 0.5% sucrose and 0.9% agar. Eight-day-old seedlings were used for chromatin extraction protocol as described previously [101]. Forty micrograms of protein samples, as estimated by by Pierce BCA Protein Assay Kit (Thermo Scientific), was loaded on 14% LiDs Tris-Tricine gels and blotted onto Immobilon-P membranes (Millipore) before immunodetection using antibodies recognizing either unmodified histone H4 (Millipore #05-858) or H3K27me3 (Millipore #07-449), H3K4me3 (Millipore #07-745R), and H3K9ac (Millipore 06-942, lot: 31636). Chemiluminescent signals were detected and quantified using a LAS4000 luminescence imager (Fuji) in three biological replicates.

### RNA-seq and differential gene expression analyses

RNA was isolated using modified TRIzol method [102] in three biological replicates for wt and *3h1* from 3-week-old seedlings pooled and frozen in liquid nitrogen. Ribosomal RNA was removed using RiboMinus Plant Kit (Thermo Fisher), and ERCC RNA Spike-In Mix 1 (Thermo Fisher) was added. Libraries were prepared with Ion Total RNA-Seq Kit v2 and Ion Xpress RNA-Seq Barcode 1-16 Kit according to the user guide. Sequencing template was generated with Ion PI™ Template OT2 200 Kit v3 on Ion OneTouch™ 2 System. Sequencing was performed on Ion PI™ chip v2 and Ion Proton™ sequencer using Ion PI™ Sequencing 200 Kit v2 (all Ion Torrent kits and software are trademarks of Thermo Fisher).

Base calling and adapter trimming were performed automatically by Torrent Suite software. Residual rRNA and ERCC reads were identified and filtered out using bbsplit and filterbyname scripts from BBTools suite [103]. Reads were aligned to TAIR10 genome using TMAP 5.0.13. with soft clipping from both ends and returning all the mappings with the best score. Other settings were set according to Torrent Suite defaults. Unaligned reads were aligned with BBMap (Brian Bushnell).

Quantitation to ARAPORT11 transcripts was performed in Partek Flow (Partek Inc.) using Partek E/M (expectation-maximization) algorithm. This allows inclusion of multi-mapping reads into quantitation. The algorithm quantitates unique alignments first and then iteratively divides the value of each multi-mapping read between possible loci based on the quantitation value in their close vicinity. The quantitation was performed on gene level (one value per gene). The read counts were normalized by the trimmed mean of M values (TMM) method [104] followed by the UQ (upper quartile) method [105]. Differential expression analysis was performed with the Partek gene-specific analysis (GSA) algorithm. It is a sophisticated version of global linear model, fitting model of variables, and theoretical distribution of expression values to each gene independently. The model was allowed to use normal and log-normal distributions to fit to the data. For log-normal distribution, the “shrinkage” method was allowed. This method, published as limma Voom [106] is specifically designed for read-based quantitative studies with low replication. It works by calculating the average trend of gene values’ standard errors in relation to average gene expression levels. Next, the errors observed for each gene are normalized towards the trend. This approach was developed to reduce the occurrence of false-positive and false-negative rate of differentially expressed gene calls, especially in low replication studies and genes with low expression, where high or low dispersion of quantitated values can result from sampling effects. Pearson correlation between replicate samples is shown in Additional file 1: Figure S10B.

To divide *Arabidopsis* genes into quintiles of expression, the quantified values from Partek E/M algorithm (described above) were normalized using reads per kilobase of million (RPKM) mapped reads method [107]. The expression values were averaged across replicates. The genes with zero values of expression in each genotype were sorted into a separate group. The remaining genes were divided into quintiles based on the expression levels separately for wt and *3h1* datasets.

Boxplots were generated with tables of RPKM values using the “boxplot” function in R language.

To calculate the distribution of genes upregulated in *3h1* versus all genes in *Arabidopsis thaliana* genome across chromatin states, genomic coordinates for chromatin state (CS) locations across the genome were downloaded from published data [40]. The genomic coordinates for genes upregulated in *3h1* were taken from TAIR 9 to be consistent with CS coordinates. Then, for each gene, the percentage of overlapped chromatin states was calculated, and for the final graph, the summary of all analyzed genes was presented.

### MNase-seq data analysis

Base calling and adapter trimming were performed automatically by Torrent Suite software. Reads were aligned with TMAP 5.0.13. Soft clipping was turned off, end repair was allowed, and all alignments for multi-mapping reads were reported. Other settings were set according to the Torrent Suite defaults. Multi-mapping read positions were resolved using MMR [108] with default settings.

Peak calling was performed on reads reaching terminal adapter with length range between 147 and 220 nt using a set of custom-made Python scripts. First read centers were piled up, and then the highest coverage positions were selected using greedy algorithm. Ends of the longest read used to define a position were used as peak boundaries. A peak was called only if its boundaries were not overlapping those of the neighboring peak. NRLs were defined by calculating peak-to-peak distances from peak calling results. The frequency of distances was calculated as a percentage of all measurements and binned into three groups. Histograms were plotted in Microsoft Excel.

Quantile-normalized wiggle occupancy files were generated with DANPOS2 [109] using the dpos function with default settings. To avoid shifting of read positions (automatic procedure for single-end reads), the program was fed with “fake 75-nt paired-end” bed files, generated from both ends of alignments of fully sequenced reads. To create fake fragments, the genomic interval of each mapped read (single-end) was divided into two 75-nt intervals on opposite strands that were used to feed the Danpos algorithm as if those were paired-end data. This did not changed the informative, real read data content but enabled to circumvent a limitation of Danpos imposing read shifting for single-end reads, which is not appropriate for single-end sequencing of full fragments after MNase digestion. Using these “fake fragments,” we could thus switch off the read shifting function in Danpos for further data processing.

Wig files were converted to BigWig format using UCSC wigToBigWig [110] and used in deepTools [111] for plotting.

Nucleosome occupancy was calculated as the number of reads, adjusted to a length of 80 nt summed over bins of 10 nt, smoothed by 20-nt running window, and quintile normalized.

#### Filtering out Ler residual sequences

Despite series of five backcrosses after introduction of *h1.3*-mutant allele from L*er* background into our *h1.1h1.2h1.3* (Col-0) line, some residual L*er* sequences were still present, mainly neighboring the *H1.3* gene. To avoid interference from those sequences in our analyses, we identified their precise genomic coordinates using SNP and coverage analyses by comparing to sequenced genome of parent L*er h1.3* line. We used those coordinates to generate bed files and filter out all reads overlapping residual L*er* sequences using bedtools intersect [112].

## Additional files

Additional file 1: Supplementary **Figures S1–S17**. (DOCX 7482 kb)

Additional file 2: **Table S1.** Quantifications of chromatin cytology corresponding to Figs. 2, 3 and 4. (DOCX 17 kb)

Additional file 3: **Table S2.** Transposable element (TE) expression in *3h1*. Available as an Excel table. (XLSX 64 kb)

Additional file 4: **Table S3, Table S5, Table S6**. Classes of TEs upregulated in *3h1*; Gene Ontology (GO) analysis of genes which are misregulated in *3h1* mutant; Expression of histone modifying enzymes in *3h1*. (PDF 903 kb)

Additional file 5: **Table S4.** Gene expression in *3h1*. Available as an Excel table. (XLSX 4453 kb)

Additional file 6: Review history. (DOCX 57 kb)

## Data Availability

The data for MNase-seq and RNA-seq discussed in this publication have been deposited in NCBI’s Gene Expression Omnibus [113] and are accessible through GEO Series accession number GSE113558 (https://www.ncbi.nlm.nih.gov/geo/query/acc.cgi?acc=GSE113558) [114]. The image data used for cytological quantifications and FRAP analyses are available at https://figshare.com/articles/3h1_vs_WT_nuclei/8223977 [115]. The Matlab-script to analyze chromatin density distribution is freely available at https://www.github.com/barouxlab/ChromDensityNano.

## Abbreviations

*3h1*: Triple H1 knockout mutant *h1.1*, *h1.2*, *h1.3*
Col: Columbia (*Arabidopsis* accession)
CS: Chromatin state
FISH: Fluorescence in situ hybridization
H1: Histone H1
H3: Histone H3
H3K27me3: Histone H3 lysine 27 trimethylation
L*er*: Landsberg *erecta* (*Arabidopsis* accession)
PRC2: Polycomb-group Repressive Complex 2
TE: Transposable Element
