# Supplementary material for: Linker histones are fine-scale chromatin architects modulating developmental decisions in Arabidopsis
Source: Genome Biol. 2019 Aug 7;20:157. doi: 10.1186/s13059-019-1767-3 (PMC6685187; doi:10.1186/s13059-019-1767-3)
Supplement: Supplementary file 1 — Supplementary Figures S1–S17. (DOCX 7482 kb) [file 13059_2019_1767_MOESM1_ESM.docx]

# Figure S1


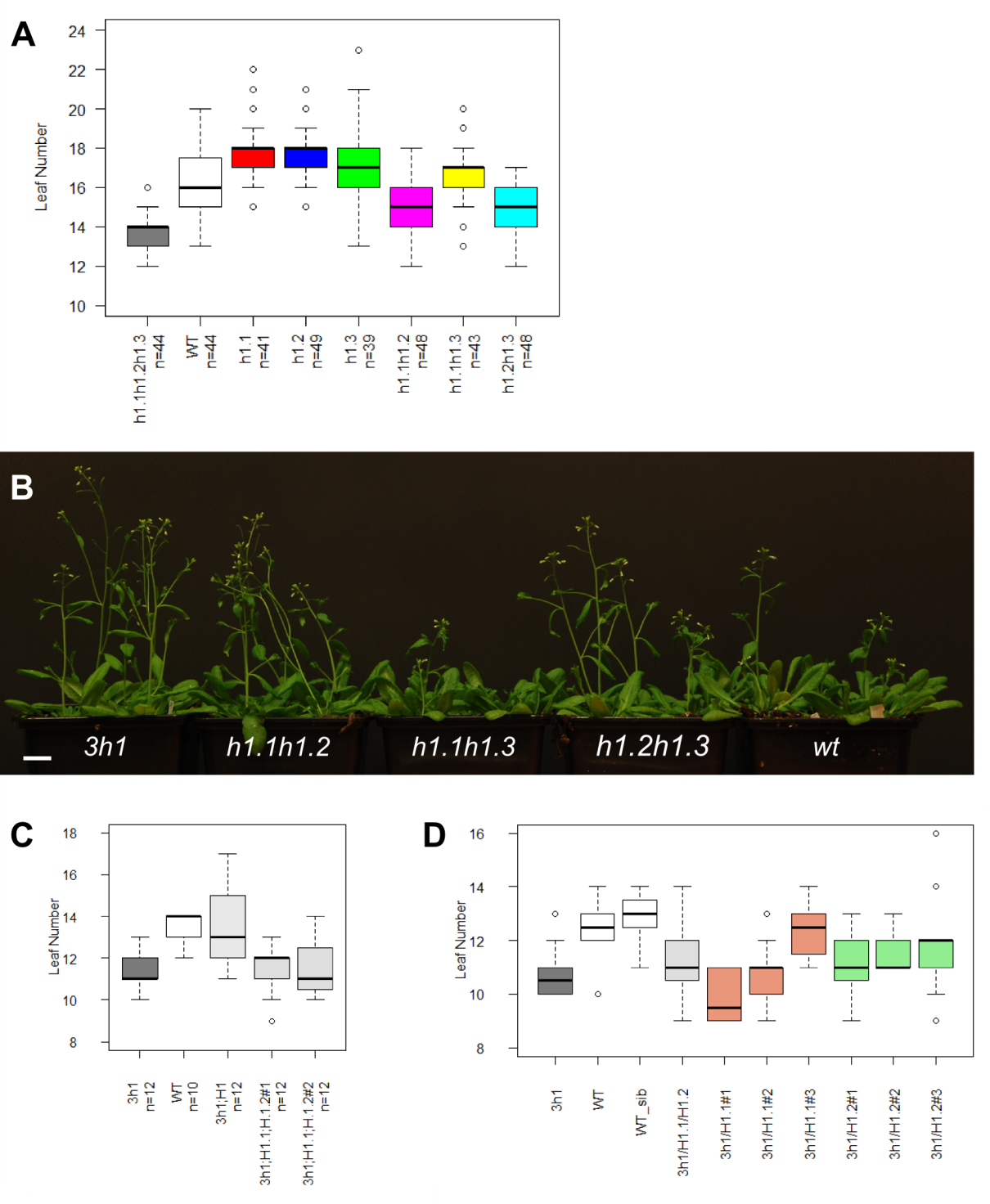


Figure S1. Flowering phenotype for *h1* mutants and complemented lines.

**D**

**(A, B)** Flowering phenotype in triple, double and single mutant combinations under long day conditions (16h day/8 h night): **(A)** Leaf number at bolting (~0.5 cm stem) and **(B)** 5-weeks old plants. For each genotype the number of 40 to 50 plants were used. The triple mutant shows an early flowering phenotype, as well as the double *h1.1h1.2* mutant albeit quantitatively less pronounced. The single mutants show normal flowering time demonstrating redundancy. *Scale bar: 2 cm* **(C, D)** Complementation assays using tagged H1.1, H1.2 and H1.3 variants: **(C)** the *3h1* early flowering phenotype is fully complemented by introducing all three H1 variants (*3h1;H1*, grey box), but poorly complemented in two H1.1 and H1.2 expressing lines (*3h1;H1.1,H1.2* lines #1 and #2, pale grey box). **(D)** in a third experiment, *3h1* was complemented with either H1.1 and H1.2 simultaneously (pale grey box), H1.1 only (red boxes) or H1.2 only (green boxes). In this experiment H1.1 and H1.2 could partially complement the early flowering phenotype but not the single variant, except for one line (*3h1;H1.1#3)* indicating a possible dosage effect.

WT= wild-type plant (Col-0), WT_sib= wild-type sibling segregants from *3h1.* The leaf number was calculated for 12 plants per genotype in (D) and “n” plants depending on genotype as indicated in the graphs (A, C).

# Figure S2


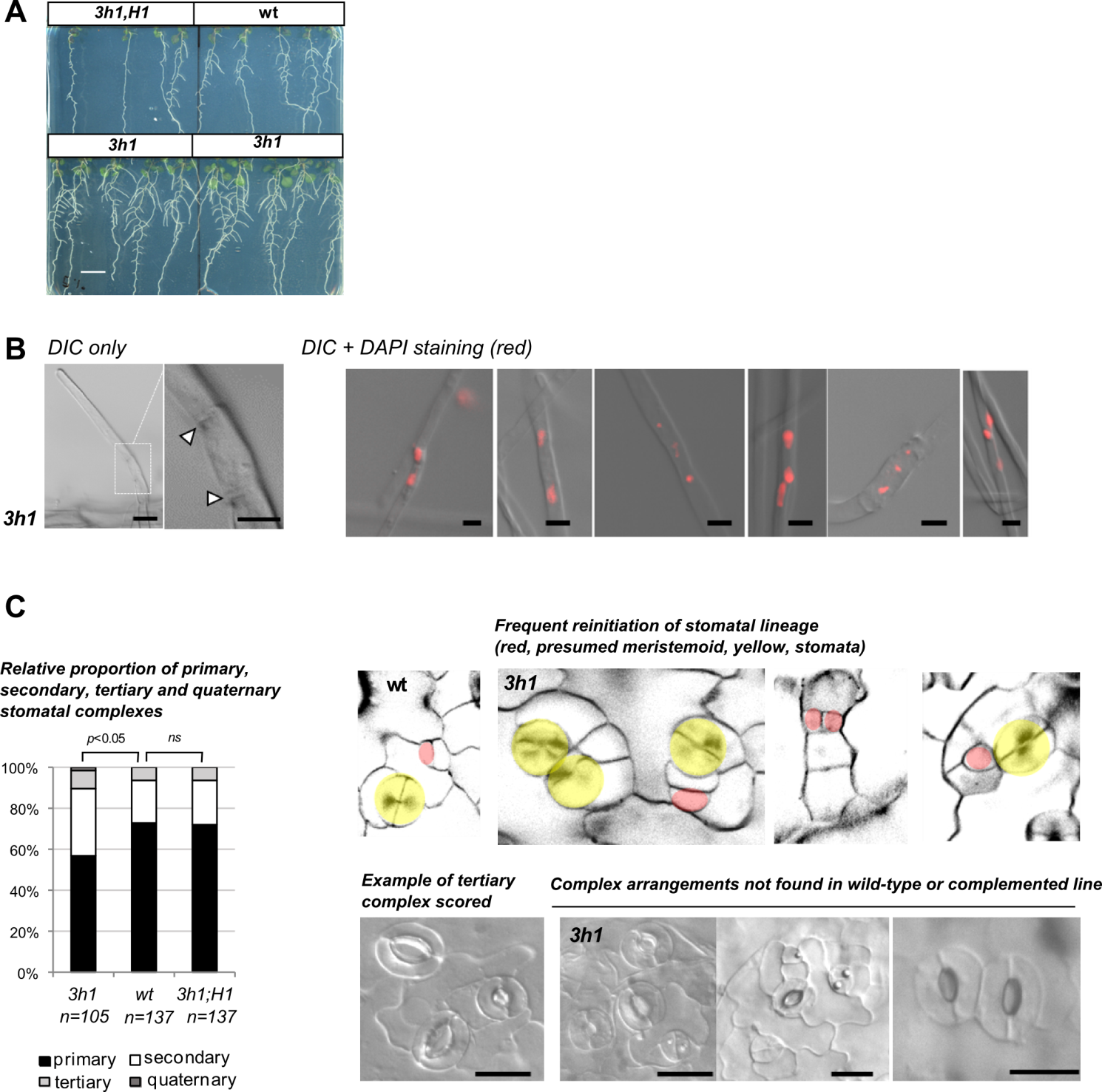


Figure S2. H1 is required for correct development of lateral root, root hair and epidermal stomata

This figure shows additional image and quantification material supporting Figure 1.

**(A)** Typical seedling phenotypes in *3h1, 3h1* complemented lines and wt showing differences in lateral root number – see Figure 1 for quantifications. *Scale bar: 1 cm* **(B)** Multicellular root hairs (see cell wall staining, Figure 1) in *3h1* mutant showing ectopic nuclei (DAPI staining, red). Multicellular root hairs were rare in *3h1* but never observed in wild-type. For each wt and *3h1* genotype, 72 seedlings (three weeks old) plated on three replicate plate, were observed under the binocular. The root hair phenotype in *3h1* was observed for 12 seedlings but not every single root hair has been inspected on each seedling, especially given the high density of root hairs in *3h1*. The seedlings were grown on MS complemented with 1% sugar under continuous light. *Scale bar: 10 µm* **(C)** Altered stomata spacing in *3h1* cotyledons generates a higher proportion of higher-degree stomatal complexes (graphs; *p-value*: χ2 contingency test; n = number of stomata complexes observed) and abnormal distributions. The images show representative examples of observations in *3h1* vs wt cotyledons upon cell wall staining (Renaissance, confocal microscopy imaging, upper panel) or without staining under DIC microscopy of cotyledon epidermal peels (lower panel). *Scale bar: 20 µm*. Seedlings 15 days after germination, adaxial side.

*Genotypes: 3h1,* triple mutant *h1.1;h1.2;h1.3*. *3h1*;*H1,* triple mutant complemented with the two H1 variants tagged with FPs*:* *3h1;prom.H1.1:H1.1-RFP; prom.H1.2::H1.2-GFP.*

# Figure S3

**
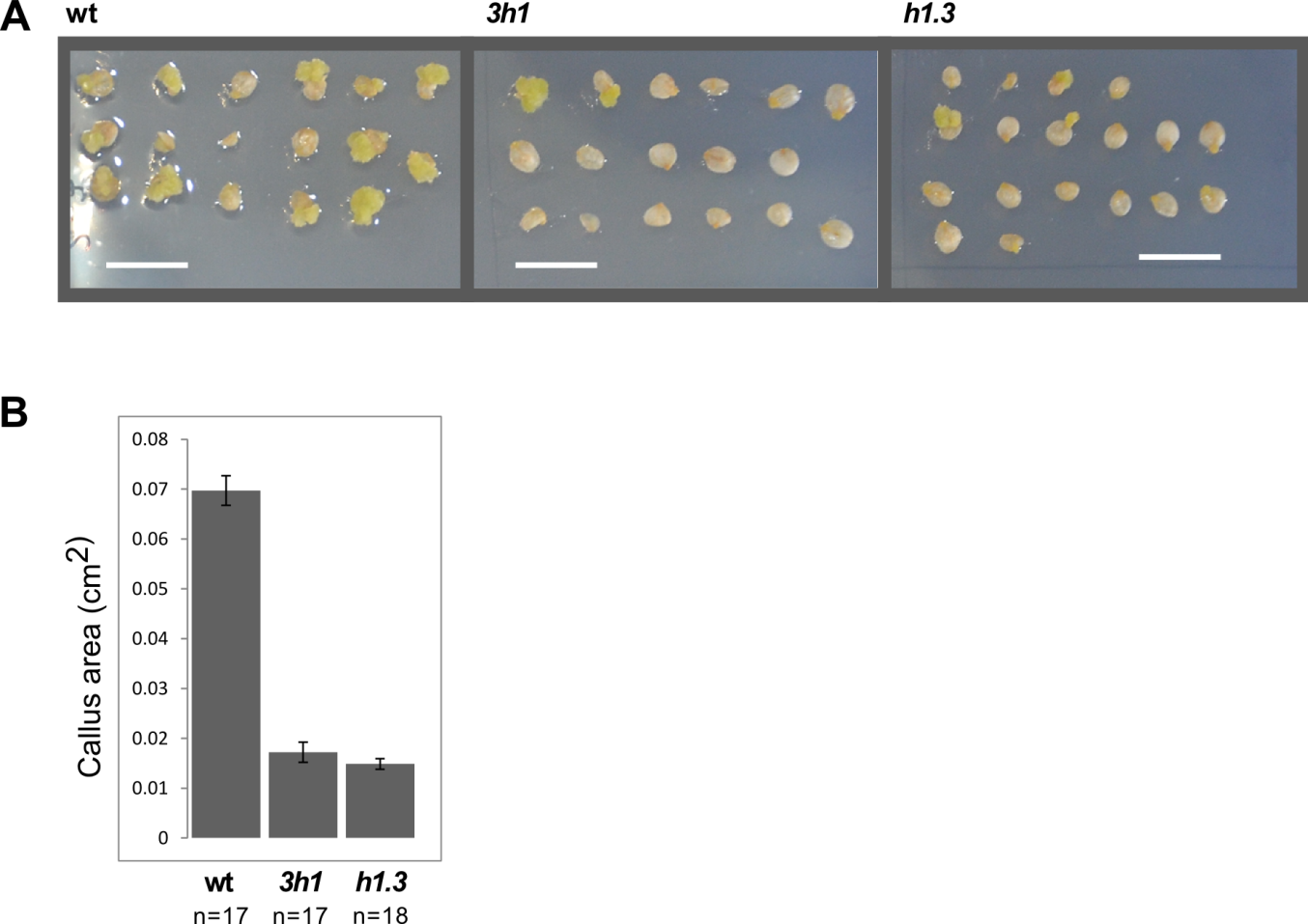
**

Figure S3. Callus formation efficiency in H1 deficient mutants is reduced.

**(A)** Comparison between callus formation in wt, *3h1* and *h1.3* 5 weeks after induction as described in the Methods. *Scale bar: 1 cm*. **(B)** Callus area (relative units) was measured for wt, *3h1* and *h1.3* with ImageJ. n, number of calli scored. The potted values are means. *Error bar: standard error of the mean (s.e.m)*.

# Figure S4


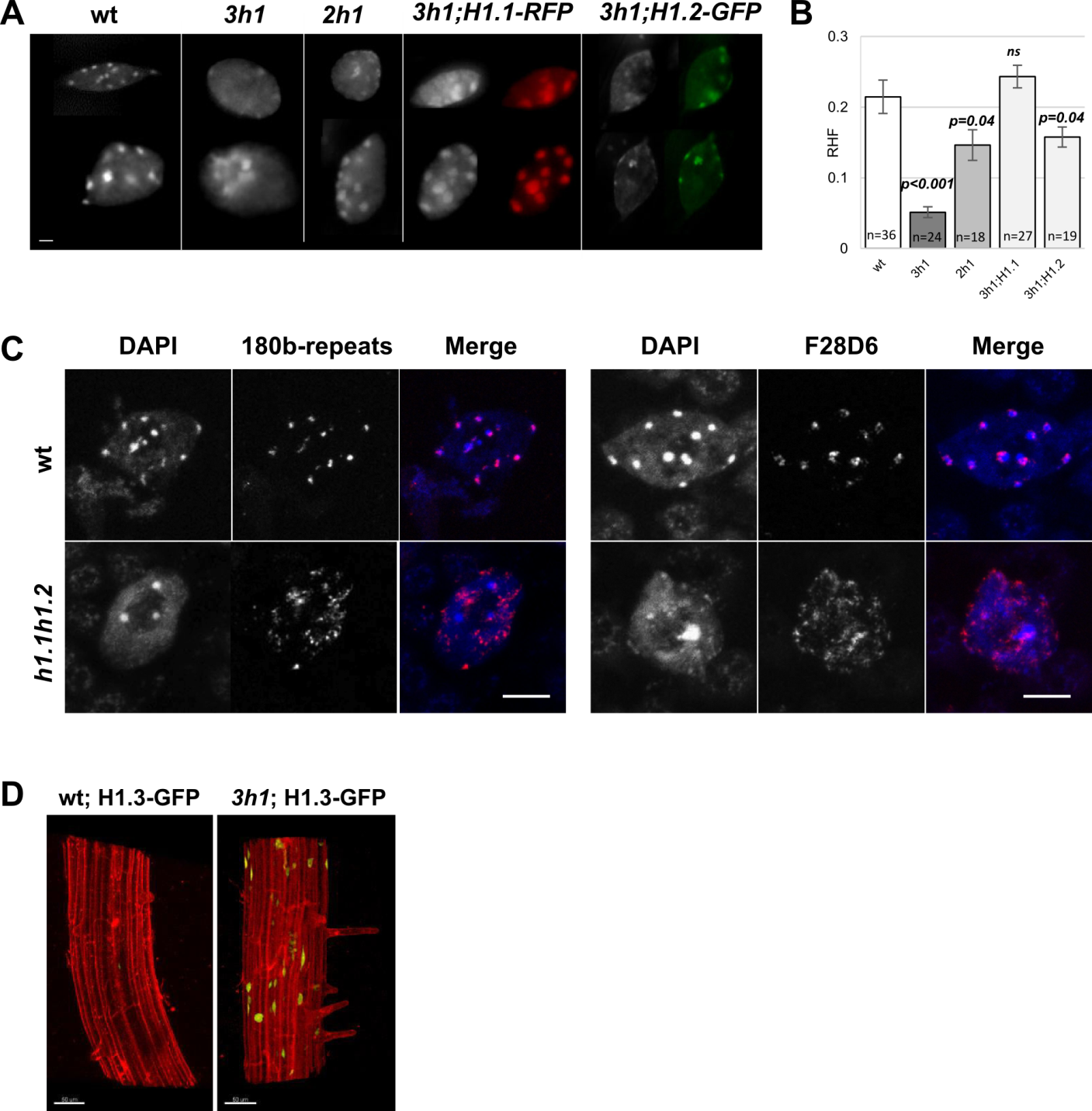


Figure S4. Compensatory effects of H1 variants in adult and embryonic tissues.

**(A-B)** Chromocenter formation in roots is dependent on H1.1 and H1.2 but not H1.3. **(A)** Typical root nuclei of 5 days-old seedling roots (in the epidermis of the differentiation zone) stained for DNA using DAPI and 1% sucrose in whole mount, in the triple mutant *h1.1h1.2h1.3* (*3h1*), double mutant *h1.1h1.2* (*2h1*), and triple 3h1 mutants complemented with either *H1.1* (*3h1;H1.1-RFP*) or *H1.2* (*3h1;H1.2-GFP*) vs wild-type (wt). Images are 3D projection from wide field epifluorescence image series. *Scale bar (the same for all images in A): 2 µm.* **(B)** Relative heterochromatin fraction (RHF) analyses from images as shown in (A) on replicate nuclei as indicated (n). The RHF loss of *3h1* measured in isolated leaf nuclei (Figure 2) is also found in root nuclei. The double *2h1* mutant shows no significant alteration. The RHF reduction in *3h1* is fully or partially restored by H1.1 and H1.2. *P-*values for t-tests against wt is indicated. *Error bar: s.e.m*. **(C)** Chromocenter formation is altered in *h1.1h1.2* mutant nuclei of embryonic cotyledons pointing out to a different chromatin composition and sensitivity to H1 loss compared to differentiated leaf or root nuclei from seedling and adult tissues. Images shows nuclei immunostained against centromeric regions with 180bp (centromeric) and F28D6 (pericentromeric) FISH probes and counterstained with DAPI. *Scale bar: 7 µm*. **(D)** Compensatory effects in *3h1* adult root tissues shown in (A) are correlated with ectopic expression of H1.3-GFP. The representative images show the early elongation zones, typical for elongated epidermal cells, in wt and *3h1* roots. As a side observation: root hairs seem to form earlier in *3h1*. *Scale bar: 50 µm*

# Figure S5


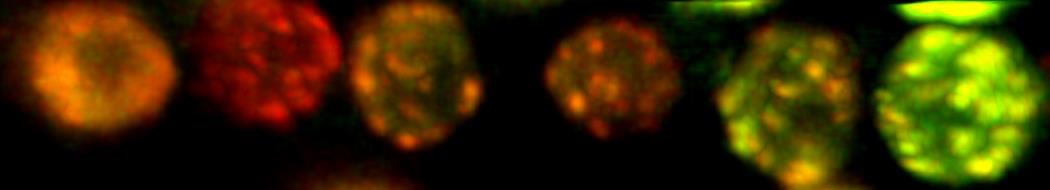


**H1.2-GFP**

**H1.1-RFP**


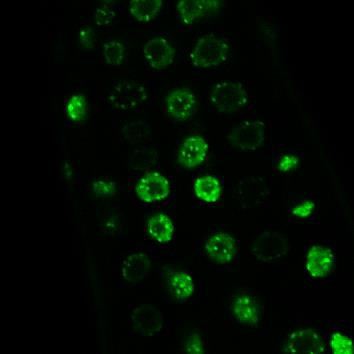

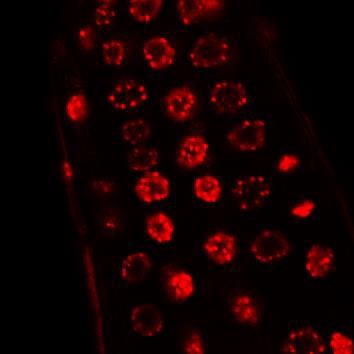

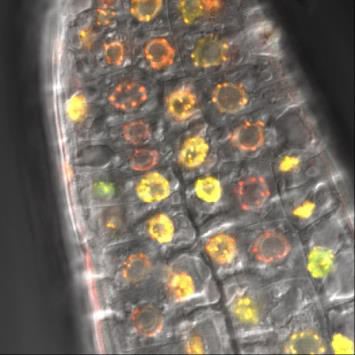


**H1.2-GFP**

**H1.1-RFP**

**A**

**B**

*differentiation*

**Figure S5. The H1.1:H1.2 relative ratio changes during cellular differentiation in root.**

**(A)** Snapshot (single plane, CSLM imaging) of a root tip co-expressing H1.1-RFP and H1.2 GFP (as indicated) in the *3h1* background overlaid with the DIC channel (right). **(B)** Representative nucleus series from a single root tip from the meristematic zone to the differentiation zone (3D projection of CSLM series).

# Figure S6


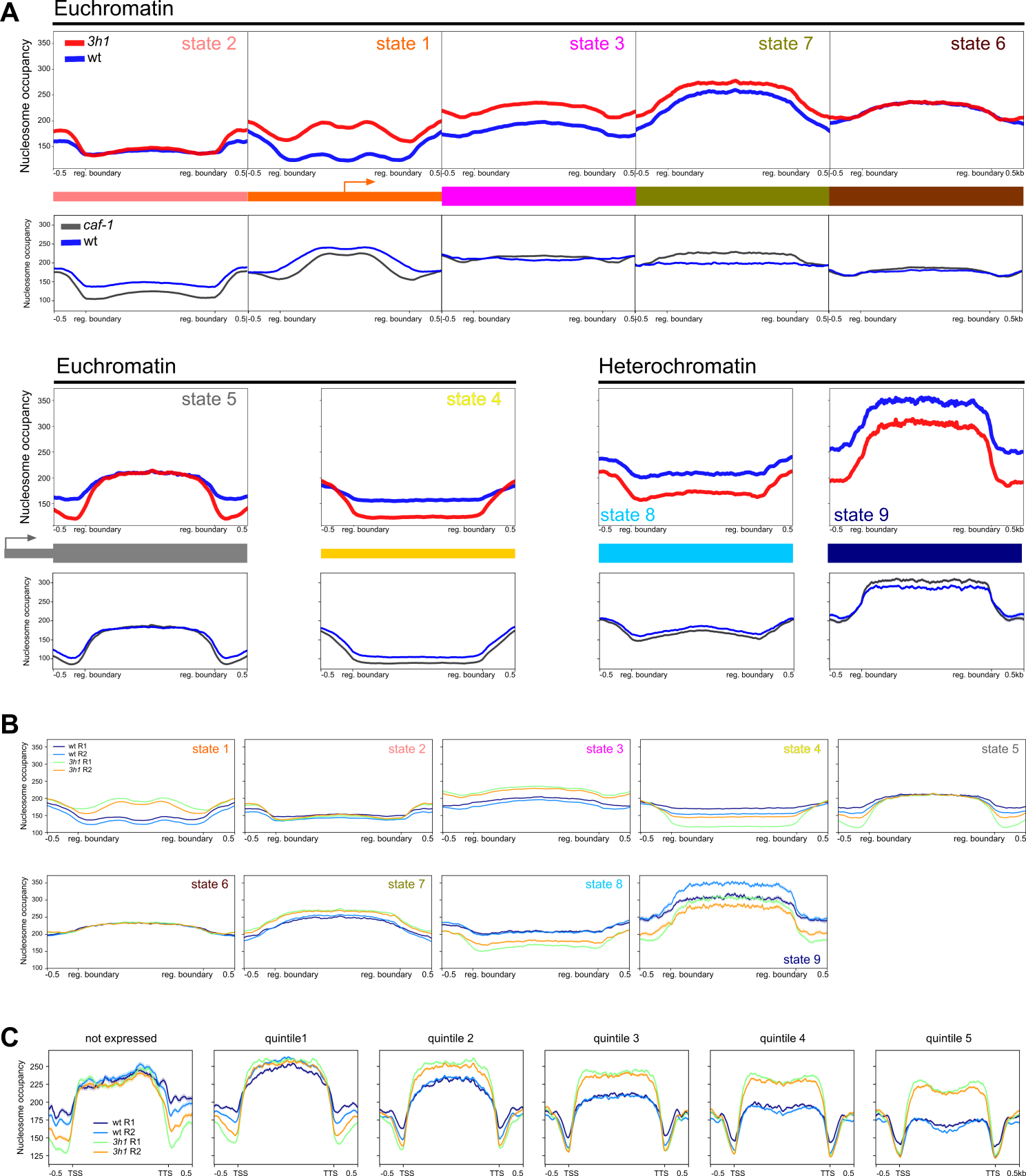


Figure S6. H1 regulates nucleosomal density with a different impact depending on chromatin states and its effect is distinct from that of the general chromatin remodeler CAF-1.

**(A)** Nucleosome occupancy quantified from MNase-seq profiles is shown per chromatin state (CS) as those defined previously (Sequeira-Mendes, Araguez et al. 2014) and after assigning each genomic regions to its more probable CS (see Methods). For ease of visualisation the CS are schematically represented along a typical coding unit, euchromatin or heterochromatin elements as proposed by Vergara and Gutierrez (Vergara and Gutierrez 2017). The plots above the genomic units show nucleosome occupancy in *3h1* compared to wild-type seedlings. The panel of plots below show nucleosome occupancy along the same units and CS in the CAF-1 loss-of-function mutant, in leaf tissue (Munoz-Viana, Wildhaber et al. 2017). The available MNase-seq data for *caf-1* and its corresponding control were processed in the same way as those generated for this study. Collectively, this analysis shows that the qualitative distribution of nucleosomes is maintained in *3h1* (red) compared to wt (blue) for each CS. However, the changes in nucleosome density at CS boundary and within the core CS element either gain (CS2, CS4, CS8, CS5) or looses (CS1) contrast in *3h1* indicating that the absence of H1 alters the segmentation of chromatin states. In contrast, loss-of-function of the general chromatin remodeler CAF-1 regulating nucleosomal assembly diminishes moderately nucleosomal density only at states 1 and 2 while not affecting the profiles. **(B)** Nucleosome coverage is shown for each replicate sample per CS. **(C)** Nucleosome coverage is shown for each replicate sample generated in this study per gene expression quantile as discussed in the text and Figure 3.

# Figure S7

**A**


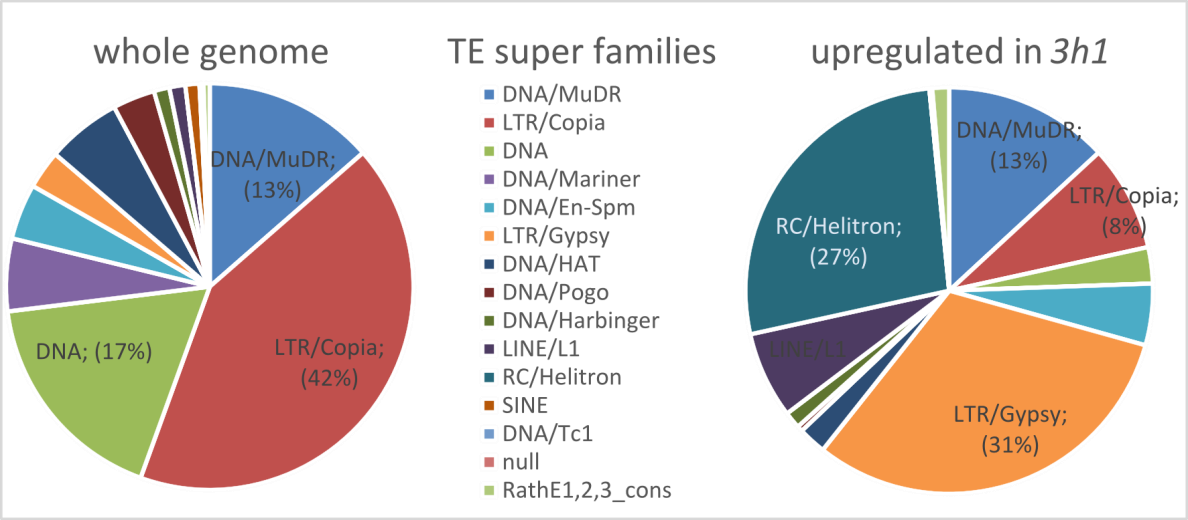


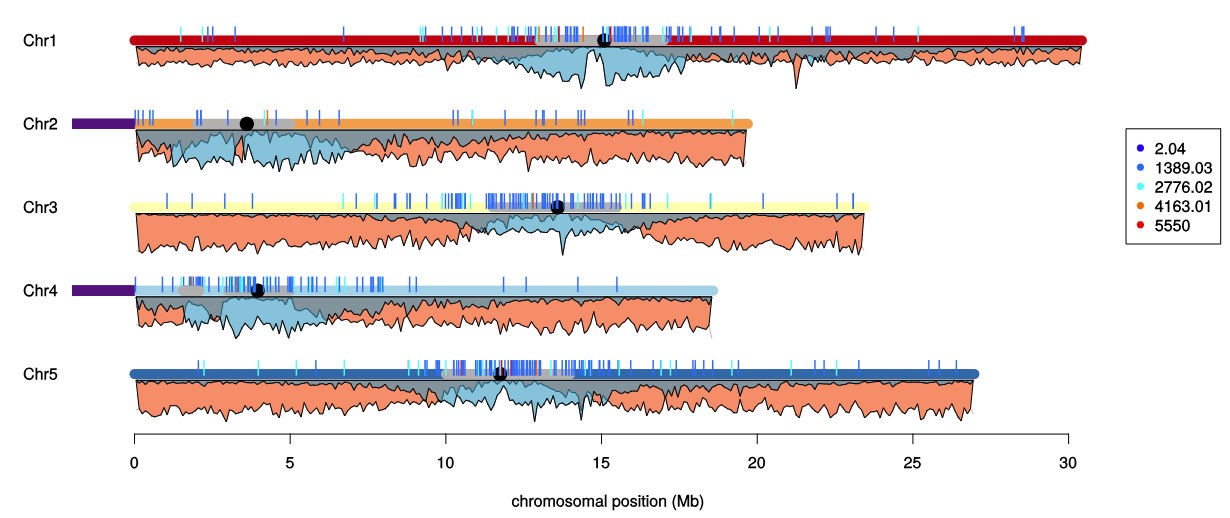
**B**

**Figure S7. Super families of TEs upregulated in *3h1* mutant.**

**(A)** The pie charts are based on the data from Table S3 (**Additional file 4: Table S3**). Upregulated elements represent only 1.5% of the all Arabidopsis TEs, are enriched in Helitron, Copia and Gypsy elements. **(B)** Distribution map of upregulated TEs in *3h1* showing mostly pericentromeric elements. The bars represent single elements, color coded for the fold change expression in *3h1*. The peak-and-valley profiles below each chromosome displays the relative enrichment in genes (orange) and TEs (blue). Graph computed in *R.*

# Figure S8


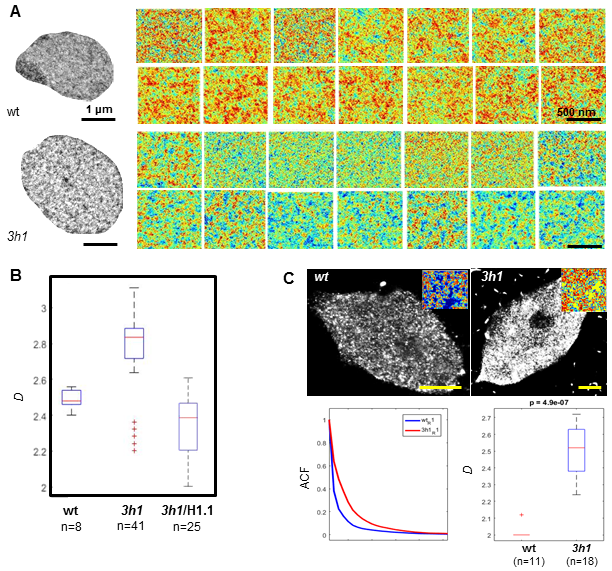


Figure S8. H1-depletion induces spatial dispersion of structural chromatin domains at the nanoscale level (complementary data to Figure 3).

**(A)** Typical TEM image of wild-type (wt) and triple mutant (*3h1*) nuclei (root epidermis) as shown in Figure 2 together with a series of representative regions of interest (ROIs) in euchromatin used for spatial autocorrelation function (ACF) analyses as described in the text and Methods. *Scale bar for images: 1 µm; scale bar for ROIs: 500 nm.* **(B)** Replicate experiment (TEM sample preparation, imaging and autocorrelation analysis) including a *3h1* mutant line complemented by H1.1-GFP (*3h1comp)* and showing the restoration of a wild-type level of the dispersion (*D*) of length scales in euchromatin. **(C)** The dispersion of nanoscale chromatin domains measured in TEM micrographs is confirmed on super resolution images (GSD imaging) of immunolabelled H3. Analysis as in Figure 2. Inset: 150 x 150 nm ROI as used for ACF analysis. *Scale bar: 2 µm*.

# Figure S9


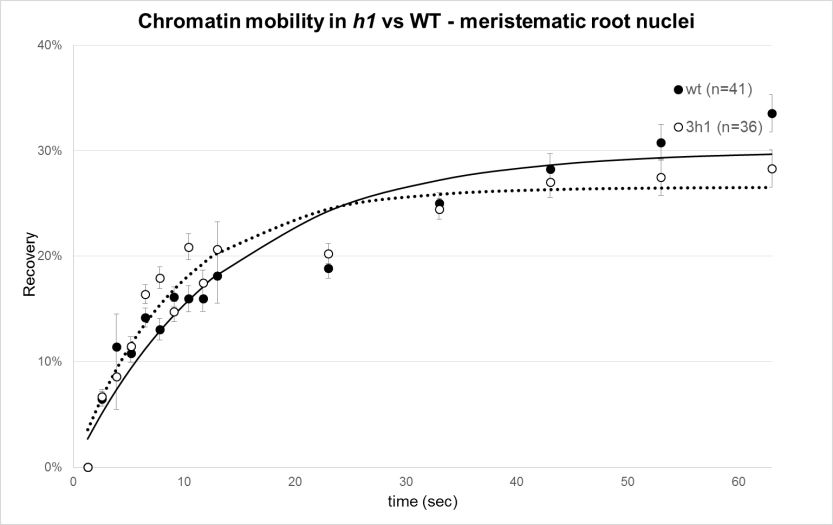


Figure S9. Chromatin mobility in meristematic nuclei is not affected by H1 depletion.

Fluorescence recovery after photobleaching (FRAP) in meristematic root nuclei in wild-type (wt) and *3h1* mutant. The graph shows a double normalisation, as done for Figure 3; see Methods. H1 depletion does not alter chromatin mobility in meristematic nuclei. Note that the recovery rate of H1-depleted differentiated nuclei (Figure 3) is similar to that of wt meristematic nuclei. *wt* are sibling segregants from *3h1* parental line. Number of analysed nuclei is indicated by “n”.

# Figure S10

**A**


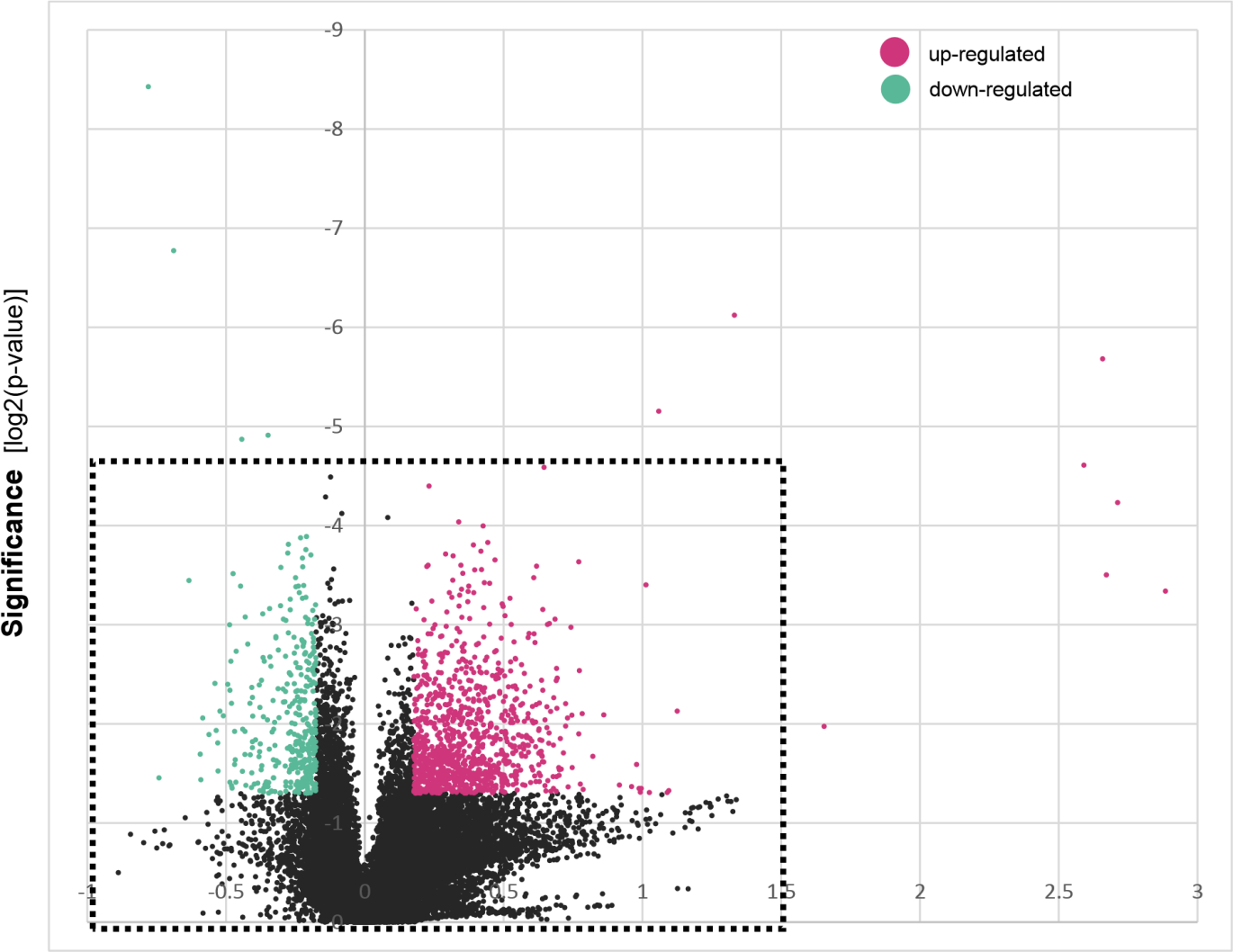


**log2(Fold change)**

**B**

|  | *3h1* R1 | *3h1* R2 | *3h1* R3 | wt R1 | wt R2 | wt R3 |
| --- | --- | --- | --- | --- | --- | --- |
| *3h1* R1 | 1 | 0.965326 | 0.968974 | 0.892405 | 0.984199 | 0.948114 |
| *3h1* R2 |  | 1 | 0.982557 | 0.951397 | 0.965333 | 0.98008 |
| *3h1* R3 |  |  | 1 | 0.933707 | 0.987749 | 0.954802 |
| wt R1 |  |  |  | 1 | 0.89707 | 0.964773 |
| wt R2 |  |  |  |  | 1 | 0.944673 |
| wt R3 |  |  |  |  |  | 1 |

**Figure S10. Gene expression changes in the *3h1* mutant compared to wild-type seedlings (full plot) and correlation analyses between replicates.**

**(A)** Volcano plot showing the significance of gene expression changes in *3h1* vs wt seedlings. Colored dots are genes significantly misregulated with a fold change of 2 or more and p-value < 0.05. This plot is the full version as the one presented in Figure 4 (cropped around the dashed box for display purposes).

**(B)** Pearson correlation coefficient between replicate samples (R1, R2, R3). The data were normalized by RPKM and log2 transformed.

# Figure S11


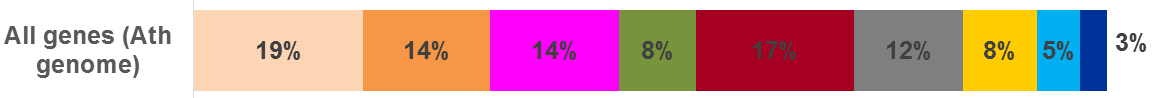

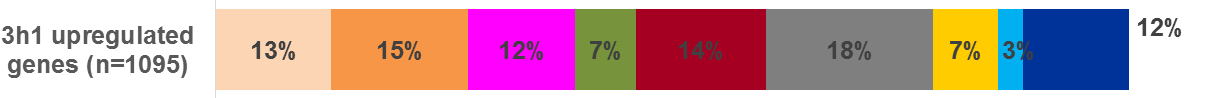


**Figure S11. Representation of chromatin states (CS) among the genes upregulated in *3h1* plants.**

The colored bars represent the relative distribution of chromatin states (CS1 to CS9 from left to right) in the *Arabidopsis thaliana* genome (gene coding regions, upper panel) compared to the distribution of CS among genes upregulated in *3h1* plants (lower panel, n=1095 genes, p-value ≤ 0.05 and fold change ≥ 1.5). Among the 1095 upregulated genes, 142 are TE genes and 12 are pseudogenes (14% collectively). This might explain the apparent overrepresentation of CS9 among *3h1* misregulated genes (lower panel). CS6 also shows a slight overrepresentation but the remaining distribution is similar to that of the genomic distribution.

# Figure S12


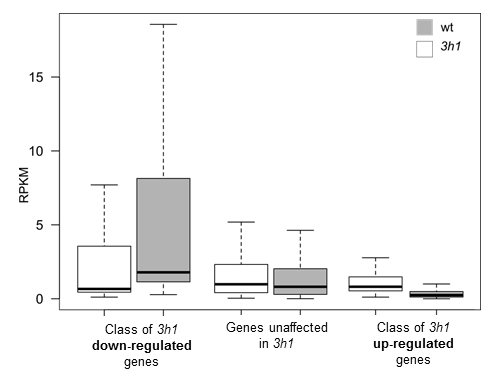


Figure S12. Up- and down- regulated genes in *3h1* correspond to gene categories with distinct expression strength in wild-type.

The graphs shows the mean expression level in RNAseq profiles for the classes of genes up- or downregulated in *3h1*, or unaffected*.* The graph shows a clear cut trend in gene classes with respect to their original expression strength in wild-type: *3h1* down-regulated genes represent a class of normally highly expressed genes in wild-type compared to the class of *3h1* up-regulated genes that represent a class of genes with low expression levels in wild-type. Average gene expression (RPKM) from 3 biological replicates for each group of genes: down-regulated in *3h1* (n=43), not-regulated in *3h1* (n=22557) and up-regulated in *3h1* (n=231); p-value ≤ 0.01 and fold change ≥ 1.5.

# Figure S13


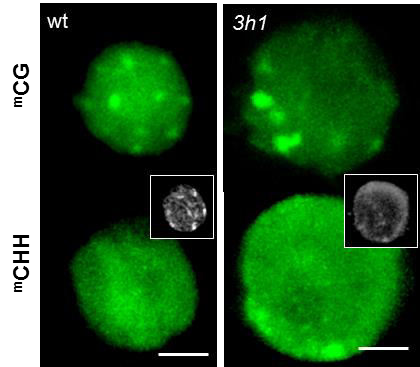


Figure S13. The nuclear distribution of CHH and CG is not drastically affected in *3h1* mutant nuclei.

Confocal imaging of root nuclei in 8 days old seedlings expressing the DynaMET reporters marking methylated DNA in the CG or CHH context as indicated (Ingouff, Selles et al. 2017). Cytological analysis of the methylation reporters do not show a significant overall reduction or massive redistribution in signal intensities in *3h1* compared to wild-type. The lower number of brightly staining foci in the mCG; *3h1* line is explained by the reduction of chromocenters in the mutant. For each wild-type and mutant lines, three independent seedlings have been analysed for distribution patterns in ca 30 nuclei per seedlings along the root (elongation to differentiation zone). *Scale bar 2 µm.*

# Figure S14
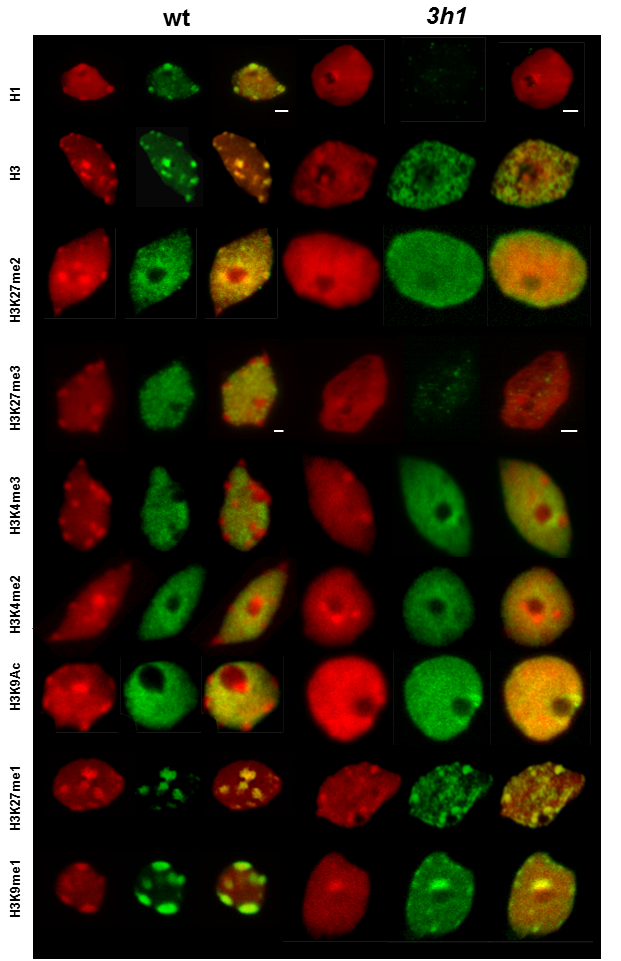


Figure S14. Representative panel of immunostained histone modifications and controls (H3, H1) in wt and *3h1* leaf nuclei.

Leaf nuclei were isolated by flow-sorting according to their 2C DNA content, fixed on slide and immunostained as described in the Methods (antibody = green; DNA counterstaining with Propidium Iodide = red). H1 and H3 immunostaining were used as controls. *Scale bar 2 µm.*

# Figure S15


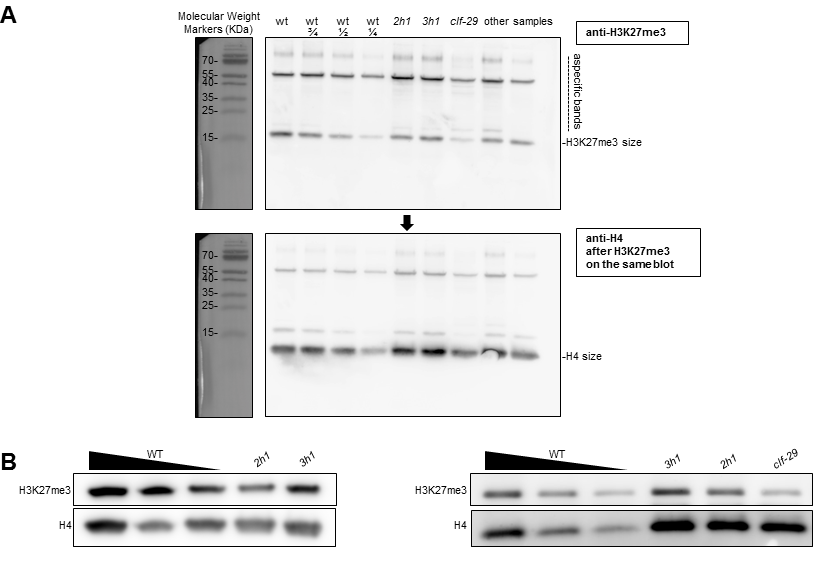


Figure S15. Global levels of H3K27me3 are reduced in whole chromatin extracts from *3h1* seedlings compared to wild-type siblings.

**(A)** Original blots and ladder used in Figure 4G. The two blots have not been cropped and modified. The same membrane was first blotted with an anti-H3K27me3 antibody and then with an anti-H4 antibody for loading control, before quantification as detailed in the Methods. **(B)** Two other independent blots used for estimating H3K27me3 signals relative to H4 in the histogram of Figure 4G. As in (A), each membrane was first blotted with an anti-H3K27me3 antibody and then with an anti-H4 antibody for loading control.

# Figure S16


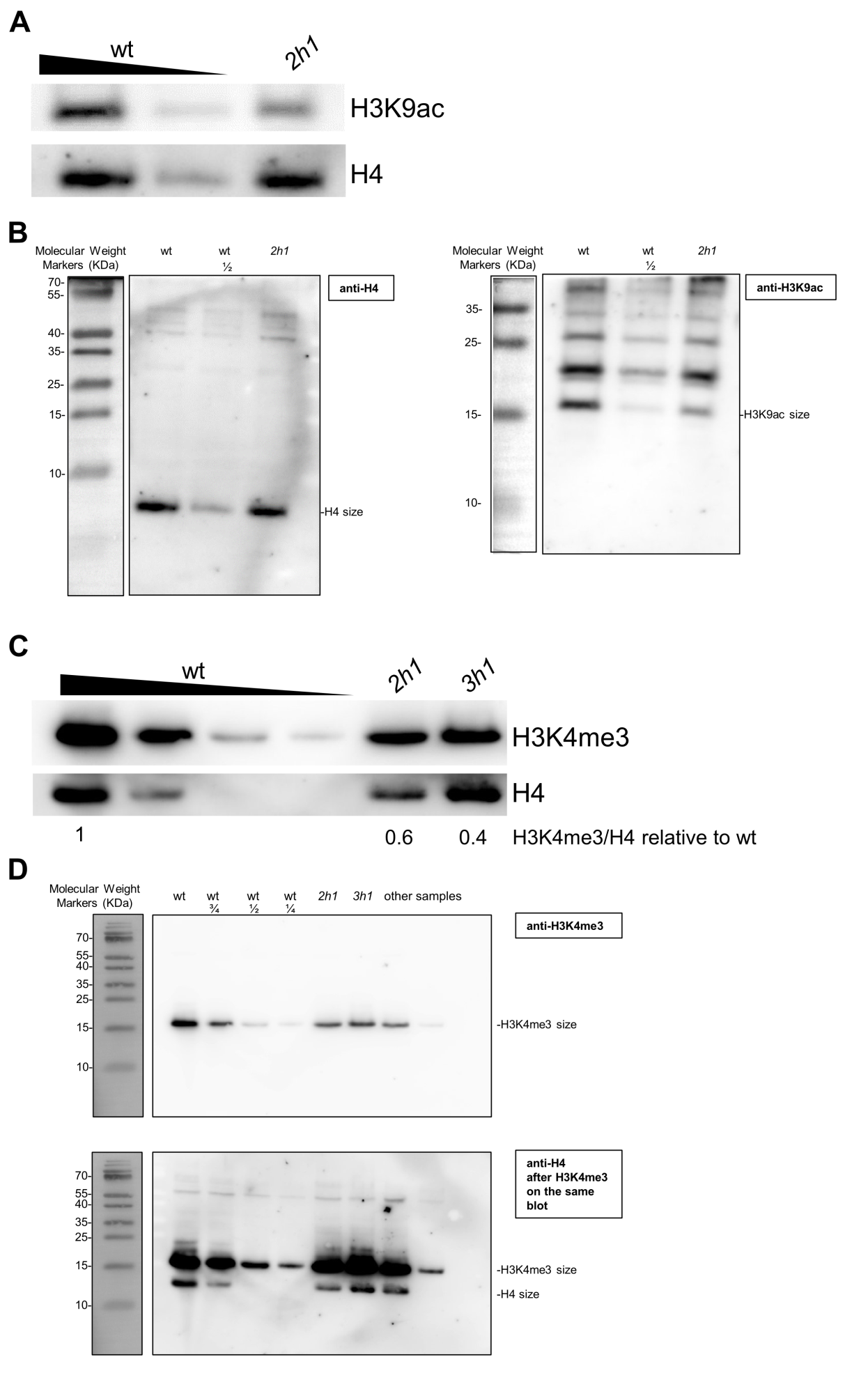


Figure S16. Confirmation of reduced of H3K9ac and H3K4me3 levels in H1-depleted tissues as seen in cytological quantifications by western blotting on whole chromatin extracts.

**(A-B)** Western Blot analysis of H3K9Ac levels in H1-depleted mutant tissue confirms the reduction seen in cytological quantification (Figure 3). Whole chromatin extracts were prepared as detailed in the Methods, blotted on a single membrane and immunostained separately with anti-H3K9ac (Millipore #06-942) and anti-H4 (Millipore #05-858) used as loading control. Two dilutions of wild-type samples were loaded (first and second lanes); the amount of the *2h1* sample (third lane, double *h1.1h1.2* mutant) was adjusted to the wild-type sample (first lane). A, cropped blots for comparison, B uncropped blots. **(C-D)** Western Blot analysis of H3K4me3 levels in H1-depleted mutant tissue confirms the reduction seen in cytological quantification (Figure 4). The membrane was first blotted with an anti-H3K4me3 antibody (Millipore #07-745R) and then with an anti-H4 antibody (Millipore #05-858) for loading control. Dilution series of wild-type samples was used for adjusting the loading of mutant samples (from *2h1* and *3h1* tissues). C, cropped blots for comparison, D uncropped blots.

# Figure S17


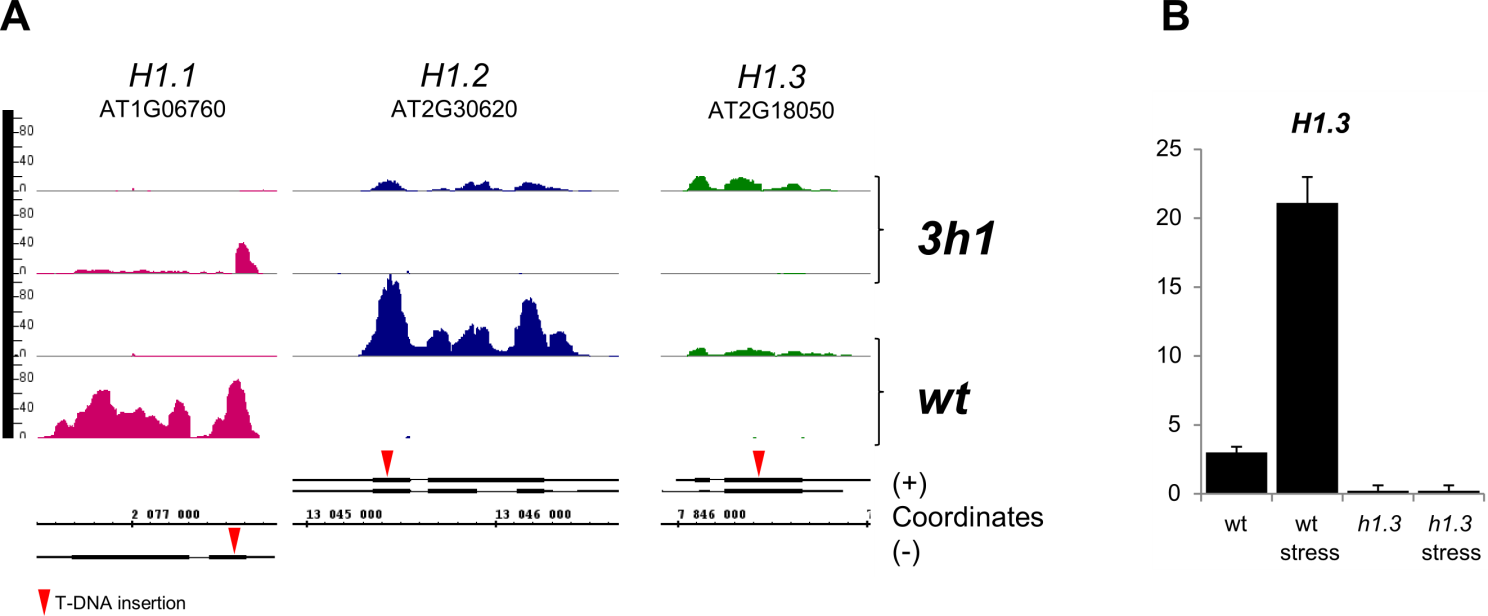


Figure S17. Characterization of *3h1* mutant.

**(A)** Local snapshots of the RNA-seq read distribution for H1 variants in wt and *3h1* mutant. Red triangle represents the site of T-DNA insertion in mutant. **(B)** RT-qPCR analysis of *H1.3* expression in control condition and after stress treatment in 3 week old wt and *h1.3* mutant plants. Stress means 4 days of decreased light intensity to ~20 µM*m^-2^*s^-1^ . RT-qPCR measurements were performed for three biological replicates and three technical replicates for each biological replicate and were normalized against the expression of Ubiquitin C (*UBC)*. Primers, detailed growth conditions, RNA isolation procedure and cDNA synthesis applied for this analysis were the same as described previously (Rutowicz, Puzio et al. 2015). The plotted values are means. *Error bar: SD*.

# References

Ingouff, M., B. Selles, C. Michaud, T. M. Vu, F. Berger, A. J. Schorn, D. Autran, M. Van Durme, M. K. Nowack, R. A. Martienssen and D. Grimanelli (2017). "Live-cell analysis of DNA methylation during sexual reproduction in Arabidopsis reveals context and sex-specific dynamics controlled by noncanonical RdDM." Genes Dev **31**(1): 72-83.

Liu, N., M. Fromm and Z. Avramova (2014). "H3K27me3 and H3K4me3 chromatin environment at super-induced dehydration stress memory genes of Arabidopsis thaliana." Mol Plant **7**(3): 502-513.

Munoz-Viana, R., T. Wildhaber, M. S. Trejo-Arellano, I. Mozgova and L. Hennig (2017). "Arabidopsis Chromatin Assembly Factor 1 is required for occupancy and position of a subset of nucleosomes." Plant J **92**(3): 363-374.

Rutowicz, K., M. Puzio, J. Halibart-Puzio, M. Lirski, M. Kotlinski, M. A. Kroten, L. Knizewski, B. Lange, A. Muszewska, K. Sniegowska-Swierk, J. Koscielniak, R. Iwanicka-Nowicka, K. Buza, F. Janowiak, K. Zmuda, I. Joesaar, K. Laskowska-Kaszub, A. Fogtman, H. Kollist, P. Zielenkiewicz, J. Tiuryn, P. Siedlecki, S. Swiezewski, K. Ginalski, M. Koblowska, R. Archacki, B. Wilczynski, M. Rapacz and A. Jerzmanowski (2015). "A Specialized Histone H1 Variant Is Required for Adaptive Responses to Complex Abiotic Stress and Related DNA Methylation in Arabidopsis." Plant Physiol **169**(3): 2080-2101.

Sequeira-Mendes, J., I. Araguez, R. Peiro, R. Mendez-Giraldez, X. Zhang, S. E. Jacobsen, U. Bastolla and C. Gutierrez (2014). "The Functional Topography of the Arabidopsis Genome Is Organized in a Reduced Number of Linear Motifs of Chromatin States." Plant Cell **26**(6): 2351-2366.
