# Supplementary material for: Linker histones are fine-scale chromatin architects modulating developmental decisions in Arabidopsis
Source: Genome Biol. 2019 Aug 7;20:157. doi: 10.1186/s13059-019-1767-3 (PMC6685187; doi:10.1186/s13059-019-1767-3)
Supplement: Supplementary file 2 — Table S1. Quantifications of chromatin cytology corresponding to Figs. 2, 3 and 4. (DOCX 17 kb) [file 13059_2019_1767_MOESM2_ESM.docx]

**Additional file 2- Supplemental Table S1**

**Quantifications of chromatin cytology corresponding to Figure 2 and 3**

1. Measurements of the RHF (relative heterochromatin fraction), chromocenters (CC) and nuclear area following DAPI staining on spread nuclei (leaf, flow sorted 2C) as described in Methods. *n,* number of nuclei; *sem*, standard error to mean

|  | **wt** | | | ***3h1*** | | | ***t-test*** |
| --- | --- | --- | --- | --- | --- | --- | --- |
|  | mean | ± s.e.m | (n) | mean | ±sem | (n) | P-value |
| **RHF** | 14.4% | ±0.33 | (n=185) | 4.5% | ±0.17 | (n=202) | 1.3E-92 |
| **Chromocenters** | 4.96 | ±0.15 | (n=185) | 2.17 | ±0.08 | (n=202) | 7.6E-49 |
| **Nuclear area** | 27.07 | ±0.57 | (n=113) | 31.95 | ±0.76 | (n=141) | 8.0E-07 |

1. Quantification of immunosignals for the histone modifications indicated normalized to DNA intensity (propidium iodide staining). Immunostaining and PI counterstaining as described in Methods on spread nuclei (leaf, flow sorted, 2C). . *n,* number of nuclei; *sem*, standard error to mean

b1) Antibody signal/ DNA signal ratios

|  | **wt** | | | ***3h1*** | | | ***t-test*** |
| --- | --- | --- | --- | --- | --- | --- | --- |
|  | mean | ± s.e.m | (n) | mean | ±sem | (n) | P-value |
| **H3K27me1** | 2.0 | ±0.09 | (n=33) | 2.2 | ±0.16 | (n=30) | 0.25 |
| **H3K27me3** | 2.5 | ±0.19 | (n=29) | 0.3 | ±0.04 | (n=28) | 2.8E-16 |
| **H3K4me3** | 1.9 | ±0.12 | (n=31) | 1.0 | ±0.07 | (n=32) | 8.7E-09 |
| **H3K9Ac** | 3.9 | ±0.32 | (n=35) | 2.5 | ±0.16 | (n=32) | 5.3E-04 |

b2) Antibody signal/ DNA signal *normalized* ratios respective to wild-type levels

|  | **wt** | | | ***3h1*** | | | ***t-test*** |
| --- | --- | --- | --- | --- | --- | --- | --- |
|  | mean | ± s.e.m | (n) | mean | ±sem | (n) | P-value |
| **H3K27me1** | 1.0 | ±0.04 | (n=33) | 1.1 | ±0.07 | (n=30) | 0.25 |
| **H3K27me3** | 1.0 | ±0.08 | (n=29) | 0.1 | ±0.02 | (n=28) | 2.8E-16 |
| **H3K4me3** | 1.0 | ±0.12 | (n=31) | 0.5 | ±0.08 | (n=32) | 1.5E-18 |
| **H3K9Ac** | 1.0 | ±0.13 | (n=35) | 0.7 | ±0.06 | (n=32) | 2.1E-21 |
