# Supplementary material for: Linker histones are fine-scale chromatin architects modulating developmental decisions in Arabidopsis
Source: Genome Biol. 2019 Aug 7;20:157. doi: 10.1186/s13059-019-1767-3 (PMC6685187; doi:10.1186/s13059-019-1767-3)
Supplement: Supplementary file 4 — Table S3, Table S5, Table S6. Classes of TEs upregulated in 3h1; Gene Ontology (GO) analysis of genes which are misregulated in 3h1 mutant; Expression of histone modifying enzymes in 3h1. (PDF 903 kb) [file 13059_2019_1767_MOESM4_ESM.pdf]

Supplemental Table 3. Classes of TEs up-regulated in *3h1.*

Super Family	Whole genome number of TE (%)		Upregulated in *3h1* (Fch≥2, *P*≤0.05) number of TE (%)	
DNA/MuDR	5410	(13%)	59	(13%)
LTR/Copia	1781	(42%)	38	(8%)
DNA	1829	(17%)	13	(3%)
DNA/Mariner	151	(6%)	0	(0%)
DNA/En-Spm	941	(4%)	22	(5%)
LTR/Gypsy	4181	(3%)	141	(31%)
DNA/HAT	1035	(6%)	10	(2%)
DNA/Pogo	344	(3%)	2	(0%)
DNA/Harbinger	379	(1%)	6	(1%)
LINE/L1	1447	(1%)	31	(7%)
RC/Helitron	12945	(0%)	121	(27%)
SINE	131	(1%)	0	(0%)
DNA/Tc1	95	(0%)	0	(0%)
null	16	(0%)	1	(0%)
RathE1,2,3_cons	391	(0%)	6	(1%)
**All**	**31076**	**(100%)**	**450**	**(100.0%)**

Supplemental Table S5. Gene Ontology (GO) analysis of genes which are misregulated in *3h1* mutant.

Biological process		
**GO Term**	**GO Annotation**	**p-value**
photosynthesis, light harvesting in photosystem I	GO:0009768	5.59E-20
photosynthesis	GO:0015979	2.42E-17
protein-chromophore linkage	GO:0018298	1.50E-16
photosynthesis, light harvesting	GO:0009765	1.73E-16
photosynthesis, light reaction	GO:0019684	7.89E-13
generation of precursor metabolites and energy	GO:0006091	5.51E-08
response to light stimulus	GO:0009416	6.51E-08
response to radiation	GO:0009314	1.62E-07
response to abiotic stimulus	GO:0009628	5.20E-07
chlorophyll biosynthetic process	GO:0015995	2.14222E-06
porphyrin-containing compound biosynthetic process	GO:0006779	5.9281E-06
tetrapyrrole biosynthetic process	GO:0033014	8.40871E-06
response to far red light	GO:0010218	1.65334E-05
chlorophyll metabolic process	GO:0015994	3.82708E-05
response to red light	GO:0010114	4.06136E-05
response to high light intensity	GO:0009644	0.000107761
porphyrin-containing compound metabolic process	GO:0006778	0.000114665
tetrapyrrole metabolic process	GO:0033013	0.000119558
response to blue light	GO:0009637	0.00021512
response to light intensity	GO:0009642	0.000226407
response to stimulus	GO:0050896	0.000376309
response to red or far red light	GO:0009639	0.001070904
response to low light intensity stimulus	GO:0009645	0.005522224
pigment biosynthetic process	GO:0046148	0.00574522
protoporphyrinogen IX biosynthetic process	GO:0006782	0.006858986
protoporphyrinogen IX metabolic process	GO:0046501	0.006858986
response to chemical	GO:0042221	0.025952734
pigment metabolic process	GO:0042440	0.026767756
heme biosynthetic process	GO:0006783	0.04207751
**Molecular function**		
**GO Term**	**GO Annotation**	**p-value**
pigment binding	GO:0031409	6.23E-19
chlorophyll binding	GO:0016168	1.43E-16
tetrapyrrole binding	GO:0046906	1.4425E-05
oxidoreductase activity	GO:0016628	0.025587703
**Cell compartment**		
**GO Term**	**GO Annotation**	**p-value**
photosystem I	GO:0009522	5.61E-27
plastid thylakoid	GO:0031976	5.67E-27
chloroplast thylakoid	GO:0009534	7.79E-27

thylakoid	GO:0009579	2.31E-26
photosystem	GO:0009521	1.55E-25
plastid thylakoid membrane	GO:0055035	3.20E-23
chloroplast thylakoid membrane	GO:0009535	3.41E-23
thylakoid membrane	GO:0042651	1.86E-22
photosynthetic membrane	GO:0034357	1.97E-22
chloroplast part	GO:0044434	3.96E-22
thylakoid part	GO:0044436	4.50E-22
organelle subcompartment	GO:0031984	4.79E-22
plastid part	GO:0044435	1.32E-21
plastoglobule	GO:0010287	4.78E-20
light-harvesting complex	GO:0030076	7.96E-18
chloroplast envelope	GO:0009941	8.15E-18
plastid envelope	GO:0009526	2.10E-17
chloroplast stroma	GO:0009570	2.24E-14
intracellular organelle part	GO:0044446	2.46E-14
organelle part	GO:0044422	2.81E-14
photosystem II	GO:0009523	4.52E-14
plastid stroma	GO:0009532	8.85E-14
plastid	GO:0009536	1.60E-13
chloroplast	GO:0009507	1.24E-12
envelope	GO:0031975	6.97E-12
organelle envelope	GO:0031967	7.04E-12
membrane protein complex	GO:0098796	1.38E-10
membrane	GO:0016020	6.96E-09
cytoplasmic part	GO:0044444	1.12E-08
cytoplasm	GO:0005737	5.33E-07
macromolecular complex	GO:0032991	8.12E-07
photosystem I reaction center	GO:0009538	2.05082E-06
protein complex	GO:0043234	0.000623038
photosystem II antenna complex	GO:0009783	0.00338651
membrane part	GO:0044425	0.006706641
integral component of membrane	GO:0016021	0.007946083
chloroplast thylakoid membrane protein complex	GO:0098807	0.008141248
intracellular ribonucleoprotein complex	GO:0030529	0.013895558
ribonucleoprotein complex	GO:1990904	0.013895558
intrinsic component of membrane	GO:0031224	0.016362585
cell periphery	GO:0071944	0.017709796
cell-cell junction	GO:0005911	0.019623792
cell junction	GO:0030054	0.019623792
cell part	GO:0044464	0.019966572
plasmodesma	GO:0009506	0.020005906
symplast	GO:0055044	0.020005906
cell	GO:0005623	0.022080537
chloroplast photosystem II	GO:0030095	0.022823861

non-membrane-bounded organelle	GO:0043228	0.022958631
intracellular non-membrane-bounded organelle	GO:0043232	0.022958631
cytosol	GO:0005829	0.023594558
organelle	GO:0043226	0.025609296
intracellular organelle	GO:0043229	0.025722053
nucleolus	GO:0005730	0.026025793
ribosome	GO:0005840	0.026106284
cell wall	GO:0005618	0.026886069
external encapsulating structure	GO:0030312	0.026886069
chloroplast membrane	GO:0031969	0.031620021
membrane-bounded organelle	GO:0043227	0.036306654
intracellular membrane-bounded organelle	GO:0043231	0.03641433
plastid membrane	GO:0042170	0.036481998
apoplast	GO:0048046	0.041207145
plasma membrane	GO:0005886	0.0462473
cytosolic ribosome	GO:0022626	0.047006336

Supplemental Table S6. **Expression of histone modifying enzymes in *3h1*.**

Gene name	Gene ID	Total counts	P-value (*3h1* vs. wt)	Ratio (*3h1* vs. wt)	Fold change (*3h1* vs. wt)
**HDAC - histone deacetylases**					
HDA19	AT4G38130	4.55E+00	9.77E-01	1.00E+00	1.00E+00
HDA6	AT5G63110	1.97E+00	9.01E-01	9.52E-01	-1.05E+00
HDA7	AT5G35600	1.03E-01	1.73E-01	6.79E-01	-1.47E+00
HDA9	AT3G44680	1.15E+00	9.63E-01	1.01E+00	1.01E+00
HDA5	AT5G61060	2.75E+00	3.82E-04	7.34E-01	-1.36E+00
HDA15	AT3G18520	1.60E+00	9.33E-01	1.07E+00	1.07E+00
HDA18	AT5G61070	1.19E+00	9.88E-04	4.55E+00	4.55E+00
HDA2	AT5G26040	8.62E-01	9.97E-01	9.90E-01	-1.01E+00
HDA8	AT1G08460	1.52E+00	4.71E-01	8.86E-01	-1.13E+00
HDA14	AT4G33470	2.91E+00	8.39E-01	9.46E-01	-1.06E+00
HDA10	AT3G44660	na	na	na	na
HDA17	AT3G44490	na	na	na	na
HD2A	AT3G44750	1.55E+00	6.90E-01	9.54E-01	-1.05E+00
HD2B	AT5G22650	3.73E+00	1.48E-01	1.21E+00	1.21E+00
HD2C	AT5G03740	2.75E+00	3.77E-01	8.92E-01	-1.12E+00
HD2D	AT2G27840	9.24E-01	3.98E-01	1.17E+00	1.17E+00
SRT1	AT5G55760	5.31E-01	5.03E-01	1.15E+00	1.15E+00
SRT2	AT5G09230	8.57E-01	8.07E-01	9.38E-01	-1.07E+00
**HAT - histone acetyltransferases**					
HAC1	AT1G79000	1.12E+01	4.58E-01	9.02E-01	-1.11E+00
HAC2	AT1G67220	2.24E+00	3.02E-04	2.25E+00	2.25E+00
HAC4	AT1G55970	1.45E+00	9.84E-01	9.78E-01	-1.02E+00
HAC5	AT3G12980	6.24E+00	3.78E-01	8.99E-01	-1.11E+00
HAC12	AT1G16710	7.25E+00	5.38E-01	9.37E-01	-1.07E+00
HAF1	AT1G32750	1.01E+01	5.33E-01	1.03E+00	1.03E+00
HAF2	AT3G19040	6.40E-01	6.91E-01	1.37E+00	1.37E+00
HAG1	AT3G54610	6.16E-01	1.13E-01	1.44E+00	1.44E+00
HAG2	AT5G56740	1.46E+00	8.46E-01	9.74E-01	-1.03E+00
HAG3	AT5G50320	2.15E+00	9.48E-01	1.01E+00	1.01E+00
HAG4	AT5G64610	2.31E+00	3.37E-01	1.04E+00	1.04E+00
HAG5	AT5G09740	9.90E-01	8.52E-01	1.01E+00	1.01E+00
**HMT - Histone methyltransferases**					
CLF	AT2G23380	na	na	na	na
SWN	AT4G02020	4.41E+00	6.76E-01	1.06E+00	1.06E+00
MEA	AT1G02580	2.49E-01	3.12E-01	1.68E+00	1.68E+00
ASHH1/ SDG28	AT1G76710	8.92E-01	7.30E-01	9.14E-01	-1.09E+00
ASHH2/ SDG8	AT1G77300	9.83E+00	7.30E-01	1.05E+00	1.05E+00

ASHH3	AT2G44150	1.05E+00	9.66E-01	9.80E-01	-1.02E+00
ASHH4	AT3G59960	1.36E-01	3.66E-01	1.07E+00	1.07E+00
ASHR3/ SDG4	AT4G30860	7.48E-01	1.69E-02	1.74E+00	1.74E+00
ATX1	AT2G31650	1.38E+00	6.82E-01	9.13E-01	-1.10E+00
ATX2	AT1G05830	3.95E+00	3.65E-01	8.97E-01	-1.11E+00
ATX3	AT3G61740	1.50E+00	1.12E-01	1.37E+00	1.37E+00
ATX4	AT4G27910	2.09E+00	1.90E-01	1.26E+00	1.26E+00
ATX5	AT5G53430	2.30E+00	3.77E-01	8.18E-01	-1.22E+00
ATXR3	AT4G15180	1.30E+01	2.51E-01	9.10E-01	-1.10E+00
ATXR7	AT5G42400	5.34E+00	4.30E-01	1.09E+00	1.09E+00
ATXR5	AT5G09790	2.83E-01	5.80E-01	1.21E+00	1.21E+00
ATXR6	AT5G24330	2.12E-01	3.72E-01	1.56E+00	1.56E+00
SUVH1	AT5G04940	2.28E+00	8.73E-01	1.01E+00	1.01E+00
SUVH2	AT2G33290	1.45E+00	4.39E-02	1.58E+00	1.58E+00
SUVH3	AT1G73100	2.22E+00	9.09E-01	1.01E+00	1.01E+00
SUVH4	AT5G13960	8.94E-01	9.20E-02	1.54E+00	1.54E+00
SUVH5	AT2G35160	1.05E+00	5.64E-01	1.22E+00	1.22E+00
SUVH6	AT2G22740	na			
SUVH7	AT1G17770	2.53E-01	3.59E-01	2.00E+00	2.00E+00
SUVH8	AT2G24740	na			
SUVH9	AT4G13460	4.29E+00	6.34E-01	9.75E-01	-1.03E+00
SUVR1	AT1G04050	7.16E-01	6.49E-04	3.15E+00	3.15E+00
SUVR5	AT2G23740	na			
SUVR2	AT5G43990	1.12E+00	7.54E-01	9.31E-01	-1.07E+00
SUVR3	AT3G03750	7.91E-01	5.08E-01	7.98E-01	-1.25E+00
SUVR4	AT3G04380	6.30E-01	3.96E-02	1.94E+00	1.94E+00
**H3K27me3 demethylases**					
ELF6/JMJ11	AT5G04240	5.44E+00	6.49E-01	9.59E-01	-1.04E+00
REF6/JMJ12	AT3G48430	6.18E+00	6.97E-01	1.06E+00	1.06E+00
JMJ13	AT5G46910	1.29E+00	4.13E-01	1.24E+00	1.24E+00

Genes showing "na" entry correspond to regions which were filtered out due to the Ler residual sequences around H1.3 locus. See Methods for more details.
