# Supplementary material for: Linker histones are fine-scale chromatin architects modulating developmental decisions in Arabidopsis
Source: Genome Biol. 2019 Aug 7;20:157. doi: 10.1186/s13059-019-1767-3 (PMC6685187; doi:10.1186/s13059-019-1767-3)
Supplement: Supplementary file 6 — Review history. (DOCX 57 kb) [file 13059_2019_1767_MOESM6_ESM.docx]

Review History

**First round of review**

**Comments to author and authors’ point-by-point responses to the reviewers’ comments:**

**Reviewer 1**

Reviewer comment: “This manuscript reports on the characterization of Arabidopsis mutants deprived of histone H1. The authors first described examples of multiple phenotypic defects of the 3h1 mutant plants. Then, they performed cytological analysis of heterochromatin organization and demonstrated that H1 was necessary for heterochromatin assembly but dispensable for silencing and nuclear peripheral positioning of chromocenters. Using MNase-seq, they showed that deprivation of H1 affected nucleosome spacing and distribution along the genome. Their RNA-seq analysis revealed that a fraction but not all of the transposon genes within heterochromatin were reactivated and that only about 700 genes within euchromatin were misregulated in the 3h1 mutant plants. Their microscopy analyses showed that H1 is present in euchromatin regions, forms nanoscale-chromatin domains, and has an inhibitory effect on chromatin mobility. Lastly, the authors showed that H3K27me3 levels were reduced in the 3h1 mutant, suggesting a link between H1 and PRC2 function but the underlying mechanism remains unclear.”

*Authors response: We have indeed shown a correlation between H1 function on chromatin organisation and abundance of H3K27me3, a chromatin mark deposited by the PRC2 complex. We first show this by cytological experiments. This was also confirmed by western blots, whose quantifications indicate a significant reduction as compared to histone H4 abundance that reflect nucleosomal content in the chromatin samples. It is to be noted that a similar diminution of H3K27me3 has been reported in H1-depleted animal cells (Fan et al, 2005). Thus, not unique to plants. We also show that the subtle phenotypes of 3h1 correspond to developmental transitions normally regulated by PRC2. Thus, our observations suggest a functional link between H1-mediated chromatin structure and PRC2-controlled processes or PRC2 activity. We agree that the mechanistic basis underlying the altered H3K27me3 levels in H1-deficient plants is not characterized here. We refer in the text to a study showing a substrate preference of EZH2 to H1- containing nucleosomes that provides a plausible scenario for our observations (Martin et al, 2006, JBC 281). Demonstrating that the plant PRC2 also shows substrate preference for plant H1-containing nucleosomes or that H1 is required for PRC2 activity involves long-term investigations that are beyond the scope of this study. We revised the corresponding statements in the abstract and rewrote the last discussion paragraph, opening a discussion on the possible role, formulated as hypotheses, of H1 in epigenetic reprogramming, not focused on PRC2 function.*

Reviewer comment: “The work was performed with very rich different experimental approaches. Nevertheless, the novelty of findings is limited to plant.”

*Authors response: We do not see this as a limitation. Eukaryotes are represented by several supergroups including those representing plants and animals. Studying chromatin organization in eukaryotes other than insect or mammalian cells offers a valuable opportunity for discussing the evolution of nuclear organization and their fundamental principles. Numerous studies reporting on the functional characterization of nuclear organisation in plants have recently been published in Genome Biology and other highimpact journals not bound to plant models. This trend provides evidence for a collective interest in expanding our knowledge to eukaryotes other than animals.*

Reviewer comment: “The described functions of H1 are consistent with previous knowledge in plants and in other organisms.”

*Authors response: The precise function of H1 on fine scale chromatin organization has never been described in plants so far. Specific studies cited in our discussion report on DNA methylation changes and the heterochromatinization potential of H1 (with experiments done mostly in tobacco). In addition, we highlight similarities but also differences with animal (mammalian, insect) cells. For instance, the concept that H1 is dynamic at, or influences, cellular transitions in adult, somatic tissues is unique to plants so far. Furthermore, as explained above, a detailed analysis in plant cells offers the great opportunity to address evolutionary questions about linker histones function and chromatin biology among eukaryotes.*

Reviewer comment: “Many conclusions drawn within the manuscript corroborate previous studies, making the novelty not very strong.”

*Authors response: It is not clear for us which previous studies are meant here. We duly cite and discuss former work showing the influence of H1 on DNA methylation (Wierzbicki and Jerzmanowski 2005, Zemach et al 2013), the potential of H1 to induce heterochromatinization in a heterologous system (Prymakowska-Bosak et al, 1996). We now cited the work of Lyons and Zilberman (2017) deepening the analysis of H1 role on DNA methylation discussed in the context of nucleosome remodeling mechanisms. So far, however, no other published study addresses the role of H1 on nuclear organization or on fine-scale chromatin structure at the cytological and molecular scale, which on its own represent a significant novelty. Moreover, no other study reports a cross-analysis with gene expression and plant phenotypes. Thus, it is not clear to us why the novelty of our work is called out in this comment.*

Reviewer comment: Specific points:

Reviewer comment: “1) Some sentences expressing strong general interests of this work in the Abstract are not fully in agreement with knowledge described in the manuscript body text.”

*Authors response: We were not entirely sure to understand which of the sentences the reviewer refers to. Nevertheless we acknowledged the possibility that several concepts were not clearly presented. Accordingly, we modified the abstract substantially to refer to the findings described in the main text while leaving speculation for the discussion.*

Reviewer comment: “Several previous publications have reported some H1 functions in plants, it is overstated that 'what their (H1) functional relevance to plant development is unknown'.”

*Authors response: In the previous version we cited the work from Zemach et al (2013), and work from the Jerzmanowski lab (co-author of this manuscript) (Wierzbicki and Jerzmanowski (2005), Rutowicz et al (2015)) describing the effect of H1 on DNA methylation. We also discussed the fact that H1 downregulation by means of RNA interference as done by Wierzbicki and Jerzmanowski (2005) produced plants with a strong pleiotropic phenotype that we do not observe with the triple knock-out mutant, and provide a possible explanation for it (off target down regulation of other GH1 protein encoding genes). We also mention the work from Prymakowska-Bosak et al (1996) showing the heterochromatinization potential of H1 in a heterologous plant system.*

*What we had, however not cited previously, is the work from Lyons and Zilberman (2017) further showing that H1 is as impediment to DNA methylation in the absence of a chromatin remodeler (DDM1), particularly at a subset of heterochromatin loci with well positioned nucleosomes. We now cite it in the revised version page 4 with the following sentence “H1-containing nucleosomes are thought to create a barrier to DNA methyltransferases, a structural conflict that can be resolved by the activity of SWI/SNF types of chromatin remodelers (Zemach et al, 2013; Lyons and Zilberman, 2017) and page 13.*

Reviewer comment: “'H1 depletion relaxes developmental transitions usually controlled by Polycomb-group repressive complex 2 (PRC2)' is also overstated.”

*Authors response: Please see our response to the first comment addressing this concern.*

Reviewer comment: “2) The description of the 3h1 mutant plant phenotype is fragmented into 'examples'. Together with previous report on 3h1, the incomplete description of mutant defects occupies reader's thinking.”

*Authors response: We acknowledge the fact that much remains to be done to propose a unifying scenario between observations at the molecular, cytological and organismal levels. Nevertheless, we believe that our study constitutes a ground-work towards this goal: it opens avenues of investigations to elucidate the link of H1 functions at the chromatin level with the developmental phenotypes. Here, we investigated the development of 3h1 mutants in detail, going much beyond a simple growth analysis. We unraveled unsuspected consequences of H1 depletion on developmental transitions that are landmarks of epigenetic switches in the plant’s life cycle (seed dormancy, flowering, the somatic-to-reproductive transition) and during cell fate establishment associated with organogenesis (in leaf and root tissues). After reviewer 1 comment, we revised the last paragraph of the discussion and believe that this now offers a plausible scenario for H1 functions while drawing future routes of investigation.*

Reviewer comment: “3) The data shown in Fig 4F need improvement.”

*Authors response: We apologize for inappropriate presentation of the Western Blot in the former Figure 4F. This figure panel being overall heavy, we had tried to minimize the area presenting the western-blot but this turned out to be misleading, especially if the reader is not aware of Figure S15 showing the full blot. The revised figure now shows un-cropped blots, which were formerly given in Figure S15, as detailed below. Reviewer comment: “The H2B panel intrigues questions about the similarity of size and different intensity of bands between wt and mutants,” Authors response: The size of the different H2B bands was actually the same as it could be seen on the intact un-cropped blot in the former Figure S15. The H2B cropped panel was however not aligned properly in the former Figure 4F. The new figure and additional blots in the supplemental Figure S15 now answers this concern.*

Reviewer comment: “also it seems there is an additional band needing blot to be shown more in full.”

*Authors response: All blots are now shown in full in Figure S15. The additional band below H2B (~18KDa) in the former Figure 4F corresponded to histone H3 (~17KDa) detected on the same membrane. Indeed, the former Figure 4F presented chromatin extracts loaded in parallel on two independent gels: the first membrane being probed successively for detecting H3K27me3 and H4, the second membrane being probed for H3 and H2B. Considering that this was largely confusing, notably because H2B and H3 were blotted on a different membrane than H3K27me3 and H4, the H2B/H3 blot has been omitted in the revised Figure 4F and S15. This blot is actually un-necessary for the conclusion as we can directly compare H3K27me3 levels to histone H4 upon quantification on the very same membrane (~12KDa for H4 versus ~17KDa for H3K27me3).*

Reviewer comment: “Other issues concern quantification. It is unclear whether wt has been in a same or a different blot that of the 3h1 and clf-29 mutants. If not, the quantification is unlikely valid. If yes, it is necessary to verify reproducibility.”

*Authors response: We concur with reviewer 1: loading all samples on the same gel and blotting them together is indeed essential for comparison. This is the case in Figure 4F, and also in the different replicates shown in Figure S15. Additional explanations on quantification are now provided in the Methods. More precisely, to ensure a proper quantification, we now rely on 1) co-migration of all chromatin samples showing proportionality of the visible signal using a wild-type dilution series, 2) immunoblotting of H3K27me3 and H4 as loading control on the same membrane, 3) quantification of H3K27me3/H4 relative levels using a luminescence imager on non-saturating signals, 4) quantification of mean levels in 3 independent biological replicates (shown in Figure S15).*

Reviewer comment: “4) Genome-wide profiling of nucleosome positioning and distribution had been reported previously.”

*Authors response: Genome-wide profiling of nucleosome positioning and distribution has not been previously reported in the triple (h1.1, h1.2, h1.3) H1 mutant. However, profiles are available for the double h1.1, h1.2 mutant (Lyons and Zilberman, 2017). As we show in our work, the H1.3 variant tends to be induced ectopically in the absence of H1.1 and H1.2. Hence our profiling study is not redundant and in addition can unmask possible confounding effects resulting from ectopic expression of H1.3 in the double mutant. Furthermore, our analyses have a different focus than those presented in Lyons and Zilberman (2017): we analyse nucleosome occupancy in relation to (i) chromatin states (C1-CS9 as proposed in other studies referenced in the text) and (ii) gene expression strength (per quintiles, as proposed in other studies), which has not been made before and is essential to link H1 function to transcription.*

Reviewer comment: “Some comparison of reproducibility (of wt) and effects of 3h1 with mutants defective in nucleosomal histone deposition (e.g. caf-1) are useful.”

*Authors response: With “comparison of reproducibility” we understand that the reviewer would like to see the independent replicates? We show in a new panel of supplemental figure S6 the independent wild-type and 3h1 replicates for nucleosome profiling along chromatin states (B) and gene expression quintiles (C). To address the request on the need to test other mutant plants with a known impact on nucleosome distribution, we now provide the analysis of MNase-seq profiles in the CAF-1 loss-of-function mutant impaired in nucleosomal histone deposition. We used MNase-seq data available (Munoz-Viana et al, 2017) using the same bioinformatics pipeline used for 3h1 analysis. The analysis shows that the caf-1 mutation alters*

*moderately the density, but not the qualitative profile, at some chromatin states (CS) but not all. As such it shows that H1 depletion has a very specific (vs generic) effect on nucleosome distribution. We now comment on it in the result and discussion sections as follows: - Results p9 after “However, nucleosomal density is clearly affected in 3h1 chromatin. Comparative density profiles for each chromatin state showed enhanced, or diminished average density levels relative to the element boundaries, as for instance the CS1 and H3K27me3-marked CS4 states, respectively (Fig. 3G, Additional file 1: Figure S6). We added “This is interpreted as a partial loss of structural differentiation of the chromatin states in the absence of H1. As comparison we re-analysed the impact of the loss-offunction of the CAF-1 chromatin remodeler on nucleosome density distribution across these CS. The analysis showed some alteration in global levels for some but not all CS states but without modifying the profile amplitudes as H1 depletion does (Additional file 1: Figure S6).” -Discussion p15 “Notably, H1 depletion affects the structural differentiation, in terms of nucleosomal density, of chromatin states. This effect is distinct from that of a loss-offunction of a general chromatin modifier such as CAF-1 (Additional file 1: Figure S6).”*

**Reviewer 2**

Reviewer comment: “The manuscript by Rutowicz, Lirski et al. reports on the functional characterization of H1 linker histones and their involvement in the modulation of chromatin organization in plants. Using an elegant combination of quantitative imaging, cytological and molecular approaches, the authors convincingly show that histone H1 is required to achieve heterochromatin compaction into chromocenters and for homogeneous organization of the euchromatic compartment. At a finer resolution, depletion of H1 globally affects nucleosome occupancy and the deposition of histone H3 modifications (H3K9Ac, H3K4me3, H3K27me3). The authors also show that depletion of the three H1 variants leads to subtle developmental phenotypes that suggest a specific role for H1 in regulating cellular transitions. Overall, the manuscript is well written, based on well-designed experiments and rigorous data analysis. The multiscale analysis proposed represents a strong asset that distinguishes this study from previous reports and is of potential interest to a broad audience. Nonetheless, a number of matters need to be properly addressed. Major points: - Despite the originality of the work, this multi-level characterization of H1 functions in plants rests on the aggregation of complementary bits of information that should be better connected with each other in order to propose a more straightforward story and draw firmer conclusions. There seems to be a disconnect between the order in which the results are presented in the title, abstract, introduction and discussion (from cytological, to molecular, to developmental) and in the results part (from developmental, to cytological and molecular), as if the developmental part was put at the beginning in a last minute decision. If developmental aspects come first, then cellular and molecular questions are expected to be addressed within one or a few relevant biological contexts affected in 3h1. If developmental aspects come last, then they may provide an appealing perspective to the general molecular and cytological observations. Given that a variety of cell types and tissues were used, the second scenario would sound more logical.”

*Authors response: We understand the concerns of the presentation choice. Accordingly, the pre-print version of this study that we deposited on BioXriv has a presentation order inverted compared to the one here. This change results from a former manuscript evaluation that suggested the actual presentation. Thus the choice may relate to subjective preferences, while the information content remains the same in both cases. In this revised version we rewrote the abstract to better match the order in the main text. We also rewrote the last discussion paragraph (also in response to reviewer 1 comment), which we believe helps rounding up the conclusions and prospective roles of H1-mediated chromatin organisation in epigenetic-based regulation of developmental transitions.*

Reviewer comment: “Anyhow, a direct connection is actually missing between the nuclear/molecular measurements and the developmental aspects. Could some of the 701 misregulated genes be associated somehow with one of the developmental defects observed in 3h1?”

*Authors response: The set of misregulated genes does not show enrichment in a specific gene category/function. Yet, transcriptome analysis was done using seedlings, i.e. in bulk tissues, an analysis that does not allow capturing the relevant developmental regulators. Answering the question of whether developmental regulators are transcriptionally affected in the mutants will require new transcriptome profiling done at specific developmental stages or on specific cell types. While this is beyond the scope of this study it constitutes a promising opening for future work. At this stage, the RNAseq analyses presented in this manuscript allowed a general investigation, which was not carried out so far, on gene expression status in plants lacking linker histones. Through this approach we unraveled that while gene coding regions are largely unaffected by the lack of H1, H1-mediated chromatin structure is epistatic to transcriptional regulation for a small number of genes. This finding sheds light on causal relationships between chromatin structure and gene expression.*

Reviewer comment: “Are some of the cytological defects, such as chromocenter decondensation, observed in most cell types?”

*Authors response: Chromocenter decondensation in 3h1 was detected in leaf (main figure) and root (supplemental figure) tissues of adult plants, as well as cotyledons of germinated seedlings (supplemental figure). It is also visible in ovule tissues as shown in supplemental data of our former work in She et al (2013). Thus the effect seems robust throughout plant tissue and development. However, cell-type specific variations in the severity of chromatin phenotypes cannot be excluded. Reviewer comment: “At the specific cellular transitions affected in 3h1?” Authors response: Heterochromatin dynamics was shown at the transition to flowering (Tessadori et al, 2007), during in vitro transdifferentiation – a step preceding callus generation (Tessadori et al, 2007) and after the release of dormancy (Van Zanten et al, 2011). Given the corresponding alterations of 3h1 plant development presented here, a role for H1 in these examples of heterochromatin dynamics is a plausible scenario. We now discuss it in the last discussion section. In addition, we attempted to analyse heterochromatin content in nuclei from cells engaging in lateral root or root hair cell formation but faced the difficulty to track the cellular transitions unambiguously both spatially and temporally. These do not occur in a predictable manner. In addition, in the absence of evident regulator affected directly by H1, providing functional evidences for a role of H1 in developmental transitions will require developing cell-specific H1 complementation experiments, a long task that goes much beyond the scope of this study.*

Reviewer comment: “- It is advisable to avoid oversimplifications in the text in order to prevent overstatements. Please amend the text throughout with respect to the following points:”

*Authors response: We acknowledge this, and thank the reviewer for pointing out inadequate formulation. We revised the text accordingly as detailed below.*

Reviewer comment: “> Heterochromatin assembly (p.8, l.120; P.16, l.310 e.g.) usually refers to the formation or reformation of heterochromatin following replication. Based on the data provided, the process affected in 3h1 seems to be primarily the condensation of some heterochromatic domains into chromocenters within interphasic nuclei. In addition, only part of the whole heterochromatin is present in chromocenters. As a result, RHF measurements only provide Relative information about the condensed Heterochromatin Fraction and not about the overall "heterochromatic content" (p.9, l.147).”

*Authors response: Regarding the term "heterochromatin assembly": this term has been used to describe cytologically-defined domains in previous studies (Soppe et al, 2002). Nevertheless, we appreciate the distinction made by reviewer 2 and changed the text accordingly using heterochromatin “formation” or “condensation”. RHF: we agree with the reviewer that cytological measurements capture only a fraction of heterochromatin, which is the fraction visible as condensed domains in the nucleus. Nevertheless, heterochromatin was historically defined as compact, conspicuous and densely staining domains (Heitz, 1928) and the RHF measurement is accepted in the field as a measure of heterochromatin in this cytological sense introduced by Fransz et al, 2002 and broadly used since then. We now modified the sentence page 6 for “In order to analyze the consequence of H1 depletion on chromatin organisation at the cytological scale we quantified the following parameters in isolated leaf nuclei from 3h1 and compared them to that in wild-type nuclei : the number of chromocenters, the relative heterochromatin fraction (RHF) measuring the fraction of chromatin condensed in conspicuous foci/domains (Fransz et al, 2002) and nuclear size informing on the global compaction level of chromatin”.*

Reviewer comment: “> The imaging approaches used to generate the data presented in Fig.3B and 3H do not provide the resolution required to visualize individual nucleosomes. Thus, the distribution of H1.1-GFP and H2B-RFP dots is unlikely to correspond to a linear interspersion of H1 and core nucleosomes (as indicated p.11, l.194) but may reflect the adjacency of larger nanodomains.”

*Authors response: We agree with the reviewer that confocal images as shown in Fig. 3B or as used for FRAP analysis shown in Fig. 3H do not provide the resolution to analyse single nucleosomes. The sentence commenting on interspersed distribution is indeed not appropriate. We changed it for “Furthermore, H1.1-GFP was found to be distributed homogenously in euchromatin with, however, the occurrence of enrichment foci relative to nucleosome distribution visualized with an RFP-tagged H2B variant (Fig. 3B, inset).”*

Reviewer comment: “Similarly, the FRAP experiments done with the H2B-RFP reporter convincingly show that H2B-containing entities show increased mobility in 3h1 but it is not possible to tell that they only correspond to nucleosomal H2B (as indicated p.12 l.228), as opposed to free histone or H2B-H2A dimers, on the one hand, or nanodomains of chromatin, on the other hand.”

*Authors response: FRAP analyses report on the mobility of the fluorescently tagged protein. Fluorescence recovery at this time scale (1 min) primarily is unlikely to capture chromatin movements (if this is what the reviewer means with referring to (labelled) nanodomains). Instead, the analysis captures H2B-RFP movement either as a result of diffusion of the free component or following incorporation (exchange) in nucleosomes. While the microscopy approach cannot resolve these two forms, it is commonly accepted that the fast recovery phase (steep slope) captures a diffusional behavior while the slow recovery phase (flatter slope) captures an incorporation (binding-unbinding) event which is a slower process (Carrero et al, 2003, Methods Enz 375, 415-422, doi: 10.1016/S0076-6879(03)75026-5, eg. Fig7). The former could correspond to free histones while the latter presumably correspond to nucleosomal (chromatinincorporated) histones. To clarify, we extended the interpretation of the FRAP analysis to clarify this issue as follows: “The analysis showed a ~2.5 times faster recovery in H1-depleted chromatin compared to in wild-type nuclei (Fig. 3H). Specifically, while the diffusional component of the recovery curve (until ~10 sec) appeared similar in both wild-type and mutant chromatins, H2B-RFP recovery rapidly reaches the slow phase characteristic of limited binding-unbinding events in wild-type chromatin (Carrero et al, 2003). The steep and continuous recovery in 3h1 indicates that H1 depletion unleashed constraints in chromatin organisation permitting rapid turnover of H2B-RFP.” Furthermore we slightly reformulated the next sentences to talk about chromatin dynamics instead of chromatin mobility: “Furthermore, this chromatin property measured in 3h1 mutant differentiated root cells resembled that of the property of wildtype chromatin in meristematic cells (pluripotent and undifferentiated) cells.”*

Reviewer comment: “- As appropriately concluded on p.15, l.295-298, H1 depletion is unlikely to specifically cause the global decrease in H3K27me3 since the abundancy of other histone modifications does also significantly change in 3h1. Such a direct connection is also not supported by the RNA-seq analysis. The apparent stronger loss of H3K27me3 measured by immunostaining could become quite similar to that of H3K4me3 if the respective amount of each histone modification (number of genes marked or overall size of genomic space covered) was taken into account. The addition of H3K4me3 in the western blot analysis could clarify this point. Based on the data provided, the connection between H1 and PRC2 put forward in the abstract and emphasized in the discussion appears a bit far-fetched and should be toned down.”

*Authors response: The reviewer’s comment reveals an interpretation that we had not foreseen. We had no intention to claim a molecular connection between H1 and PRC2 activity. We claimed, however, a functional connection because the absence of H1 leads to alteration in the H3K27me3 landscape, which is the readout of PRC2 activity. Defining a molecular mechanism connecting H1 and PRC2 would require a novel study deploying in vitro reconstitution of a plant PRC2 complex and plant nucleosomal arrays with or without H1 to test for PRC2 activity and propagation of H3K27me3. Such a study is beyond the scope of this work. Interestingly, however, it has been shown for the animal components counterpart that EZH2 prefers H1-containing nucleosome substrates to H1-free nucleosomes (Martin et al, 2006; JBC 281, doi: 10.1074/jbc.M513425200). This study hence provides an appealing interpretation for our observation in Arabidopsis. However, as we see that our formulation was not clear we modified the corresponding statements in the abstract and rewrote the last discussion paragraph which opens a discussion on the possible role, formulated as hypotheses, of H1 in epigenetic reprogramming, not focused on PRC2 function. Regarding the second comment on the "number of genes marked or overall size of genomic space covered", we indeed plan to compare H3K27me3 genomic patterns in wild-type and 3h1 plants by ChIP-seq, a task rendered difficult by the strong decreased level in the mutant. In the revised manuscript, we already provide a western-blot quantification of both H3K27me3 and H3K4me3 levels relative to histone H4 (see details of the quantification in the response to reviewer 1). This confirmed that abundance of the two marks is affected in 3h1 seedlings as compared to a nonmodified histone reference. As requested by reviewer 2, we therefore now provide a western blot in Figure S16 showing that H3K4me3 levels are also reduced in the 3h1 mutant. Reviewer comment: “Additional points:”*

Reviewer comment: “- Please specify whether the 3h1 mutant is null for each H1 variant gene as this information is not available in the indicated reference (She, Grimanelli et al., 2013). Local snapshots of the RNA-seq read distribution at the three loci would be informative.”

*Authors response: We included local snapshots of the RNA-seq read distribution for H1 variants in wt and 3h1 mutant in Additional file 1: Figure S17A. Moreover, because H1.3 is mostly expressed in stress condition we are showing also by qPCR analysis that there is no H1.3 expression in the h1.3 mutant allele (used in 3h1) after stress treatment (Additional file 1: Figure S17A).*

Reviewer comment: “Please indicate that molecular analyses were performed in three replicates. Please mention the degree of correlation between replicates and how the replicates have been dealt with to generate the list of misregulated genes.”

*Authors response: We have now amended the corresponding method to describe the use of three replicates and the algorithm used to calculate differential gene expression using a commonly used global linear model approach taking into account the variability between wt and mutant replicates to assess differential gene expression. We also provide a table with Pearson coefficients of correlation between replicate samples in Additional file 1: Figure S10B.*

Reviewer comment: “Partek flow is a commercial, proprietary suite of tools and, irrespectively of the features afforded by this tool, the authors need to provide analytical procedures and output files that are accurate and comprehensive enough to be MINSEQE compliant.”

*Authors response: We have now amended the corresponding method to describe the analytical procedure as requested.*

Reviewer comment: “Please provide the appropriate legend to the different seq-related files.”

*Authors response: We have submitted each seq-related files to GEO as indicated in the Methods. This includes a detailed description of sample acquisition as requested by GEO. Hence all the information is available.*

Reviewer comment: “Please describe how MNase-seq raw data were converted into the nucleosome occupancy values represented in Figures 2-4 and S6.”

*Authors response: We have now amended the corresponding method to describe the analytical procedure as requested.*

Reviewer comment: “- The authors may be willing to check whether the loci misregulated in 3h1 tend to be located in chromosomal regions with frequent higher-order chromatin contacts as determined by publicly available conformation capture experiments.”

*Authors response: We believe that with “higher-order chromatin contacts”, the reviewer refers here to (probabilistic) contact frequencies measured in HiC chromosome capture experiments. Using the data from Grob et al (2013) we analyzed the variance in contact frequencies, total number of contacts, and the number of trans-chromosomal contacts per genomic bins covering the genes misregulated in 3h1. None of these parameters were significantly different from randomly sampled genomic bins (sampling 10000 times, using 50kb bins).*

Reviewer comment: “They may also check whether these loci tend to be co-regulated in response to stress or to some rhythmic environmental signal.”

*Authors response: The misregulated loci were not significantly enriched for genes annotated to be responsive to (a)biotic stresses or light, apart from the downregulated genes encompassing several genes associated with light-related metabolism (indicated in the main text). To fully answer the question as whether H1-mediated chromatin structure affects transcriptional responses to stress or rhythmic environmental cues is an ongoing study in our lab and will be the topic of a future manuscript.*

Reviewer comment: “- Epigenetically compromised mutants, such as those deficient in DNA methylation or H3K27me3, do not show necessarily massive transcriptional misregulation of all the sequences associated with the corresponding chromatin modifications. Why should it be different for H1?”

*Authors response: We agree that plants appear to buffer relatively well epigenomic alterations, as shown for several generations of DNA methylation mutants for instance. This does not apply, however, for PRC2 mutants in Arabidopsis. For instance, loss of functions of the core PRC2 component CLF and SWN severely compromise fertility and viability. Here we report that H1 depletion indeed does not massively misregulate gene expression, while, however, dramatically reducing H3K27me3 levels. One possible explanation proposed in the discussion is that, for the majority of loci, H1-mediated chromatin folding is not epistatic to transcriptional regulation. For a few loci, however, it could be the case and H1 depletion could lead to gene down or up-regulation. Whether this is a result of altered accessibility to transcriptional activator/repressor, respectively, or nuclear positioning, remains to be determined.*

Reviewer comment: “- P.13 l.253, "MNase structure" is meaningless, please modify this sentence.”

*Authors response: Thank you for pointing this out. It was corrected for “H1-mediated nucleosome occupancy” as for the first part of the statement in this sentence.*

Reviewer comment: “- P.28 l.603. Please indicate the growth conditions used to generate the root hair micrographs.”

*Authors response: Thank you for pointing this insight. The growth conditions are now indicated in the methods.*

Reviewer comment: “- For all figures, please provide the appropriate scale bar for each micrograph.”

*Authors response: Thank you for pointing out this repeated insight. The figures has now been revised for the scale bars.*

Reviewer comment: “- Figure 1: > Panel B: please indicate what the indicated numbers stand for.”

*Authors response: The ‘n=’ correspond to the number of plants analysed. We amended the legend accordingly. “(n= number of plants analysed in this replicate. More are shown in Additional file 1: Figure S1)”.*

Reviewer comment: “> Panel E&F: please indicate what the arrows point to and how many lines were analyzed.”

*Authors response: We had indicated the number of seedlings and replicate plates analysed in Additional file 1: Figure S2. But we now expanded the explanations in the legend of Figure S2 and refer to this supplemental item in the legend of Figure 1. In addition, we now simplified the panels and transferred some images in the Additional file 1: Figure S2. We added a wild-type root hair and clarified the corresponding legends regarding the arrows and cell wall staining.*

Reviewer comment: “- Figure 3B: Please indicate the physical length of the scale bar.”

*Authors response: This is now corrected. Thank you for the notice.*

Reviewer comment: “- Figure 4G: How do the 1096 genes misregulated in 3h1 relate to the 701 mentioned in the text?”

*Authors response: The difference comes from using different statistical criteria for each case. The first group used in Venn diagram to crosscheck with H3K27me3 targets was generated using p-value < 0.05. The group of 701 genes mentioned in the text was generated with more strict criteria: p-value < 0.05 and fold-change ≥ 2. Thank you for pointing this out. This criteria has now been added in the legend of Figure 4.*

Reviewer comment: “- Figure S2: > Panel B: please increase the contrast or add arrows to specify cell separations as some examples appear to correspond to cells with split nuclei.”

*Authors response: We added a close up picture in DIC showing cellular separation visible in DIC. Upon DAPI staining, the optical quality did not allow observing the separations as without staining (‘DIC only’ in the new panel) or upon cell wall staining (Renaissance, Figure 1E).*

Reviewer comment: “> Panel C: please specify the number of observations.”

*Authors response: The number of observations correspond to the “n”, number of stomatal complexes, indicated in the graph. We rearranged panels C and D into a single panel C and added images of cell-wall stained (Renaissance) epidermis showing the presumed meristemoid cells and stomata, suggesting frequent reinitiation events in 3h1 and explaining the higher frequency of high-degree stomata complexes.*

Reviewer comment: “- Figure S4: > Panel A: The two typical 3h1 nuclei show a quite different DAPI signal. It is surprising that the corresponding RHF measurements shown in panel B have such a small variability with respect to the other genetic backgrounds.”

*Authors response: We show indeed several representative examples, instead of only one, DAPI stained nuclei imaged in whole-mount roots. As noticed by the reviewer, the overall DAPI intensity sometimes vary, likely due to different endoreduplication states as commonly found in root tissues. However, the RHF expresses the fraction of heterochromatin present in the chromocenters relative to total chromatin in the nucleus. The relatively low standard error to mean shown in the graph indicates that the RHF is not dependent on the global ploidy level.*

Reviewer comment: “> Panel D: It is not clear whether the two confocal images correspond to the same root zone. What does "adult" mean?”

*Authors response: The question likely arises because the image of the 3h1 root shows well-formed root hairs while the corresponding wild-type shows only emerging structures. But indeed, both images correspond to the same root region recognizable by the elongated epidermal cells. We now clarified it with the following statement in the Figure S2 legend: “The representative images show the early elongation zones, typical for elongated epidermal cells, in wt and 3h1 roots. As a side observation: root hairs seem to form earlier in 3h1.“ (Additional file 1: Figure S2).*

Reviewer comment: “- Figure S6: the legend is difficult to understand. Please rephrase.”

*Authors response: We have now rephrased the legend. We also added the analysis in caf-1 mutant and per independent replicate as requested above. The legend has been amended accordingly.*

Reviewer comment: “- Figure S13: Please indicate the number of nuclei analyzed.”

*Authors response: We revised the legend and added “For each wild-type and mutant lines, three independent seedlings have been analysed for distribution patterns in ca 30 nuclei/seedling along the root (elongation to differentiation zone)”.*

Reviewer comment: “- Figure S14: There is no micrograph for the 3h1 H1 panel. Please add the corresponding image even if there is no signal. Add H3K27me3 and H3K4me3 immunostainings for comparison purpose.”

*Authors response: Thank you for pointing out this insight. A new set of images is now provided. As requested we also generated a gallery showing all histone modifications mentioned in this study for comparison at a glance.*

Reviewer comment: “- Table S5: Please add histone-coding genes in this table. It is not clear why some genes have no reads.”

*Authors response: We thank the reviewer for this suggestion. Providing the reads for all variants of all histone coding genes would provide a very large report (ca 50 genes in total). We think that this would not help interpreting the data here. All profiles generated in this work are publically available and can easily be inspected in browser interfaces for genes of interest. Moreover, Table S4 (Additional file 5: Table S4, sheet “all_genes”) contains information on the expression data for all Arabidopsis genes in 3h1 vs wt. Therefore we propose not to make the table S5 more complex than it currently is. The purpose of this table is to show that the expression of candidate chromatin modifiers are unaltered, particularly those involved in histone modifications for which we see alterations in 3h1. Some of the genes listed have no reads due to our specific filtering procedure of residual Ler containing regions around the h1.3 mutant locus (see Methods). We added the explanatory sentence below this table (Additional file 4: Table S6) concerning this issue: “Genes showing “na” entry correspond to regions which were filtered out due to the Ler residual sequences around H1.3 locus. See Methods for more details.”*

**Second round of review**

**Comments to author and authors’ point-by-point responses to the reviewers’ comments:**

**Reviewer 1**

Reviewer comment 1: The revised Abstract fits better with current knowledge. Yet, some sentences remain problematic, for instance what means ‘functional evolution of H1 variant’ and ‘our knowledge in plants remains sparse’? Is ‘Fine-scale chromatin analyses’ meaning cytology analysis? I am not sure that the authors’ data can draw a conclusion on H1 variants for ‘distinct roles in euchromatin and heterochromatin and a dual causality on gene expression’. Many euchromatin genes are also unaffected in the 3h1 mutant.

*Authors response: - the term “functional evolution of proteins” refer to the evolution of the function and expression of protein variants among organisms. See Catarazo et al. (2019) Functional Evolution of Proteins. Proteins 87(6): 492-501 doi: 10.1002/prot.25670. Nevertheless, we modified the sentence for “Well studied in animals, little is known about the evolution of H1 function in other eukaryotic lineages for instance plants”*

*- Fine-scale chromatin analyses refers indeed to the cytological analyses provided at a quantitative level, with high resolution imaging and ultrastructural imaging (ie Transmission electron microscopy, TEM), hence transcending simple qualitative observations and beyond conventional microscopy imaging. We left this term as it is well understood in the community.*

*- The conclusion for distinct roles of H1 in euchromatin and heterochromatin is based on the different effects of H1 depletion in (mostly) chromatin organisation in these compartments: (i) H1 depletion in heterochromatin affects mostly compaction but not epigenetic identity nor expression, albeit at 1.5% TE. (ii) H1 depletion in euchromatin by contrast, although leading to a global decompaction, ie at nuclear scale as seen by an increased nuclear volume, does not prevent local compaction at the nanoscopic scale but leads to a higher dispersion, ie less regular, distribution of nanodomains, and drastically alter epigenetic identity as seen for H3K27me3, H3K4me3, H3K9Ac (likely a maintenance issue). From profiling we also measure variable effects on nucleosome density. Thus, in our opinion this conclusion of dual role is highly justified.*

*- we explain the conclusion of “dual causality” in the text : while for a vast majority of loci, H1 depletion does not override transcriptional regulation, for a subset of loci H1 depletion impairs the proper transcriptional controls leading to up or down regulation.*

*Hence, there are two distinct causal relationships between H1 and gene expression.*

Reviewer comment 2:

While ‘H1 depletion impairs H3K27me3 level’ is quite convincing, its influence on H3K9ac and H3K4me3 remains questionable. The authors corrected from an increase to decrease for H3K9ac in revision, but this is difficult to be reconciled with data shown in Figure S14. Western-blot analysis for H3K9ac will be helpful.

*Authors response: While we acknowledge the need to scrutinise each experiment in such an evaluation process, we do not understand the qualifier “questionable” here. We provide robust cytological quantification –that are rarely done in the field, where analyses commonly remain at the qualitative level- and western blot quantification that replicate the results.*

*- The cytological quantifications are based on the analysis of 30 to 35 nuclei for H3K4me3 and H3K9Ac analyses (see main figure and supplemental tables).*

*- A single image does not allow to assess visually this decrease. While unfortunately a common practice, only “looking” at a few images to deduce levels is a wrong approach.*

*For this reason, it is important to measure immunosignal intensities using real pixel values and relate them to DNA staining signals (ie proxy to DNA content) over several nuclei. This is what we have done in this study to secure a meaningful quantification.*

*- We provided already previously a western-blot confirming the decreased level of H3K4me3 upon the reviewer request. This is thus a confirmation of this observation at the global scale. The experimental results are thus replicated independently and we do not see a reason to qualify them as “questionable” with our current knowledge of these experimental practices.*

*- We now provide a Western-blot confirming the impact of H1 depletion on H3K9Ac levels upon the reviewer’s request, although this is not the main focus of this paper.*

Reviewer comment 3: Regarding the western-blot shown for H3K4me3 (Figure S16), a decrease of H3K4me3 in the PRC2-defective mutant clf-29 is questionable. Ii’s known that H3K27me3 antagonizes H3K4me3, and several previous studies reported an increase of H3K4me3 in clf.

*Authors response: We agree that this observation was not expected. We did not intend clf as a control for quantifying this mark actually. We simply loaded the same samples (wt, 3h1, clf) as for the H3K27me3 analysis. This finding is thus serendipitous and not at all relevant for our work.*

*Nevertheless, we searched in the literature for possible precedent analyses. Unfortunately, the Reviewer is not specific about publications which supports the statement “it is known that H3K27me3 antagonizes H3K4me3, and several previous studies reported an increase of H3K4me3 in clf”. In fact, while some publication report H3K4me3 changes at specific loci (ie chosen for the study), there is hardly any global analysis of H3K4me3 levels in the single clf mutant. We identified only two works:*

*(1) Lafos et al., 2011, “Dynamic Regulation of H3K27 Trimethylation during Arabidopsis Differentiation”, PlOS Genetics.*

*> In Figure 6G, H3K4me3 levels appear at equal or a lower level in the single clf-28 mutant in comparison to wild type Col-0 plants. But certainly no increase as mentioned by the reviewer.*

*(2) Jiang et al., 2008 “Repression of FLOWERING LOCUS C and FLOWERING LOCUS by Arabidopsis Polycomb Repressive Complex 2 Components”, PlOS One.*

*> In Figure 5C, H3K4me3 levels appear at equal or a lower level in the single clf-8 mutant in comparison to wild type Col-0 plants. But certainly no increase as mentioned by the reviewer.*

*However, in both cases there is no dilution series nor proper quantifications. Thus, in contrast to published data, our experimental setup quantifying H3K4me3/H3 global levels and including dilution series is a robust analysis.*

*(3) Liu et al 2014, “H3K27me3 and H3K4me3 Chromatin Environment at Super- Induced Dehydration Stress Memory Genes of Arabidopsis thaliana” (Mol. Plant 7(3)) reports that H3K4me3 levels is not necessarily antagonizing those of H3K27me3 and the authors also comment that their result contract the common view (not substantiated) that H3K4me3 is gained upon loss of H3K27me3*

*In conclusion, it has not been demonstrated that loss of H3K27me3 in clf automatically leads to increase of H3K4me3 levels in this mutant. However, since it is definitely not the scope of our study, we remove the corresponding clf lane which was not intended as control in the H3K4me3 western blot.*

Reviewer comment4:

The inclusion of caf-1 data (Figure S6) is appreciated. However, consistencies to previous publications on nucleosome occupancy (Munoz-Viana et al., 2017; Lyons and Zilberman, 2017) are not clarified. Viewing from Figure S6, the pattern for different states (particularly state 3 and state 7) is clearly inconsistent for wt in the current study (3h1) and the former study (caf-1).

*Authors response: - we understand this comment as the wish to see a comparison of wild-type samples (since both studies refer do NOT provide profiles in the 3h1 mutant) but the aim of such a comparison is not explained by the reviewer.*

*- we are convinced that comparing our mutant samples to our own wild-type (wt) samples is the most meaningful approach for such a comparative analysis. We do not see what we would learn by comparing our profiles to other wild-type profiles from plants grown under different conditions, sampled at different age, hence possibly yielding differences not related to the genotype.*

*- we provided already previously (Figure S6) profiles for the wt samples used in the study of caf by Munoz-Viana et al., 2017. However the wt samples are different than those used in our study (caf/wt = leaf 6, 38 days old ; 3h1/wt= whole seedlings, 3 weeks old), which may explain the differences pointed out by the reviewer.*

*Investigating the developmental or environmental impact on nucleosome occupancy per state is not the focus of our work.*

*- our MNAse profiles per gene expression quantile in wild-type is similar to what was previously published and already cited in the text (Li et al 2014, doi: 10.1111/tpj.12499)*
